# Comparative Study of Effects of Different Organic Acid Treatments on Preservation Quality of Two Bamboo Shoot Species Using PCA Comprehensive Evaluation and Quality Index Models

**DOI:** 10.3390/foods15142551

**Published:** 2026-07-20

**Authors:** Yuanyuan Li, Huijuan Lu, Chang Xu, Qifan Liu, Shuang Qiu, Xuejun Yu

**Affiliations:** 1 State Key Laboratory for Development and Utilization of Forest Food Resources, Zhejiang A&F University, Hangzhou 311300, China; yuanli@zafu.edu.cn (Y.L.); huijuanlu2001@foxmail.com (H.L.); xuchang1999123@foxmail.com (C.X.); qf790345164@163.com (Q.L.); 2 Bamboo Industry Institute, Zhejiang A&F University, Hangzhou 311300, China; 3 Bamboo Shoot High-Efficiency Utilization Center, China National Academy of Bamboo Industry, Huzhou 313300, China; 4 College of Biosystems Engineering and Food Science, Zhejiang University, Hangzhou 310029, China; sq16@zju.edu.cn

**Keywords:** bamboo shoot, organic acid, postharvest, preservation quality, comparative evaluation

## Abstract

Bamboo shoots undergo rapid postharvest quality deterioration. To evaluate the effects of organic acid treatments on the quality preservation of green bamboo (*Dendrocalamopsis oldhami*) shoots (GBSs) and lei bamboo (*Phyllostachys praecox f. preveynalis*) shoots (LBSs), shoots were immersed in oxalic acid (2.5, 5, 7.5 mmol·L^−1^), ascorbic acid (28.4, 56.8, 85.2 mmol·L^−1^), citric acid (26, 52, 78 mmol·L^−1^), or salicylic acid (0.5, 1, 1.5 mmol·L^−1^) for 10 min and then stored at 4 °C for 14 days. Changes in weight loss, soluble protein, soluble sugar, lignin, cellulose, taste attributes (sweetness, bitterness, astringency, umami), phenylalanine ammonia-lyase (PAL), peroxidase (POD), and polyphenol oxidase (PPO) activities were monitored. Postharvest organic acid treatments effectively preserved the storage quality of both GBSs and LBSs by reducing weight loss; suppressing lignin and cellulose increases; maintaining higher soluble protein, soluble sugar, sweetness, and umami; and inhibiting bitterness, astringency, and PAL/POD/PPO activities. Optimal treatments were variety-specific when evaluated by the principal component analysis (PCA) comprehensive evaluation model: 5 mmol·L^−1^ oxalic acid (OA2) for GBSs, and 56.8 mmol·L^−1^ ascorbic acid (AA2) for LBSs. In contrast, 56.8 mmol·L^−1^ ascorbic acid (AA2) performed best for both GBSs and LBSs when evaluated by the quality index model. Further comparison revealed that the PCA comprehensive evaluation model provided a more accurate and discriminative assessment of preservation efficacy for different organic acids than the quality index model. Moreover, OA2-treated GBSs had a longer shelf life than AA2-treated LBSs. Consequently, by employing the PCA comprehensive evaluation model, this study demonstrated that 5 mmol·L^−1^ oxalic acid can effectively preserve bamboo shoot quality, and variety-specific organic acid selection is essential for optimal storage.

## 1. Introduction

Bamboo shoots are esteemed as a nutritious vegetable, prized for their crisp texture, unique flavor, and health-beneficial components, including dietary fiber, vitamins, and minerals. Green bamboo (*Dendrocalamopsis oldhami*) shoots (GBSs), in particular, are notable for their elevated content of potassium, calcium, and trace elements like selenium. Concurrently, lei bamboo (*Phyllostachys praecox f. prevernalis*) shoots (LBSs) are extensively cultivated for their superior taste and quality in Asian countries. However, the high commercial potential of fresh bamboo shoots is severely constrained by rapid postharvest deterioration. This process is predominantly characterized by accelerated lignification and enzymatic browning, leading to a drastic increase in firmness, loss of tenderness, and discoloration, which collectively diminish marketability and shelf life [1].

To mitigate these postharvest losses, various preservation techniques have been explored. These include physical methods such as low-temperature storage [2], modified atmosphere packaging, and gamma irradiation [3,4]; chemical treatments like melatonin [1]; and coating technologies [5]. While often effective, some of these methods face limitations regarding cost, technical complexity for large-scale application, or potential sensory alterations, underscoring the need to develop safer, more economical, and efficient alternatives.

In this context, given previous research on the use of exogenous organic acids in delaying the senescence of other fruits and vegetables [6,7,8,9,10], the application of exogenous organic acids may emerge as a promising, eco-friendly postharvest strategy for bamboo shoots. These approaches primarily utilize low pH and associated biochemical effects to slow spoilage, inhibit pathogens, and reduce quality losses, with research focusing on both direct organic acid treatments and fermentation strategies that generate acids in situ. The main acid-based preservation methods can be categorized as follows: direct application of organic acids such as oxalic, citric, ascorbic, and salicylic acid via dips or sprays to control microbial growth, browning, softening, and chilling injury [6,7,8,9,11]; lactic acid fermentation, which relies on lactic acid bacteria (LAB) to produce acid and other metabolites, commonly used in products like kimchi and sauerkraut [12,13,14,15,16]; the use of slightly acidic electrolyzed water for washing fresh-cut produce to achieve strong microbial inactivation and browning control [17]; and hurdle technologies that combine organic acids with other treatments like coatings, modified atmosphere packaging, or high-pressure processing to maximize shelf life [10,18,19,20,21,22].

The preservative action of acids is multifaceted. Firstly, organic and lactic acids exert antimicrobial effects by lowering the external and internal pH of produce and disrupting microbial cell membranes [7,8,12,14,15]. Secondly, specific acids, like oxalic and salicylic acids, can delay ripening and senescence by reducing ethylene production, respiration rate, water loss, and chilling injury [6,7,9]. Thirdly, they are effective in controlling enzymatic browning and oxidation; citric acid, ascorbic acid, and oxalic acid can inhibit browning enzymes and help maintain antioxidant levels, including vitamin C [6,7,8,21].

Organic acids, such as oxalic, ascorbic, citric, and salicylic acids, can delay senescence by suppressing ethylene biosynthesis and respiration, control browning by PPO activity, and mitigate lignification by downregulating key enzymes in the phenylpropanoid pathway, including PAL, cinnamyl alcohol dehydrogenase (CAD), and POD [6,7]. Furthermore, they can enhance the activity of antioxidant enzymes, like ascorbate peroxidase (APX) and catalase (CAT), alleviating oxidative stress and membrane damage [23]. Specifically for bamboo shoots, postharvest treatment with oxalic acid has been demonstrated to effectively retard lignification and browning during cold storage [24].

Despite this progress, significant knowledge gaps remain. First, existing research has predominantly focused on oxalic acid, leaving the efficacy of other common organic acids, like ascorbic, citric, and salicylic acids, on bamboo shoots largely unexplored and uncompared. Second, it is unclear whether the optimal organic acid treatment is universal or varies with bamboo shoot species, given their potential physiological differences. A comparative study of distinct varieties like GBSs and LBSs is lacking. Third, most evaluations have centered on visual and textural attributes, with insufficient attention paid to comprehensive nutritional (e.g., soluble protein, soluble sugar) and sensory taste profile changes (e.g., umami, sweetness, bitterness, astringency) during storage under different organic acid treatments.

Beyond traditional physical and chemical preservation techniques, the use of statistical and computational tools to analyze and predict fruit quality has become an increasingly important strategy. Advanced analytical methods, such as machine learning algorithms and multivariate statistical approaches, offer promising pathways for interpreting extensive experimental data and refining postharvest management. Mathematical normalization and integration approaches that combine all measured parameters to give a single score, such as principal component analysis (PCA) and quality index (Q_i_), are commonly adopted [25,26,27]. Such methods enable dimensionality reduction, identification of dominant variables, and discovery of latent patterns within complex datasets [28,29].

Therefore, this study aimed to systematically investigate and compare the effects of postharvest treatments with oxalic, ascorbic, citric, and salicylic acids at varying concentrations on the quality preservation of two economically important bamboo shoot varieties, GBSs and LBSs, by using a PCA comprehensive evaluation model and quality index model during cold storage. The assessment encompassed weight loss, lignification, key nutritional components, taste attributes, and the activities of related enzymes (PAL, POD, PPO). The findings are expected to identify variety-specific optimal treatments and provide a deeper physiological understanding, thereby contributing to the development of targeted, effective, and green preservation strategies for the bamboo shoot industry.

## 2. Materials and Methods

### 2.1. Plant Materials, Treatments, and Storage

**Figure 1:**
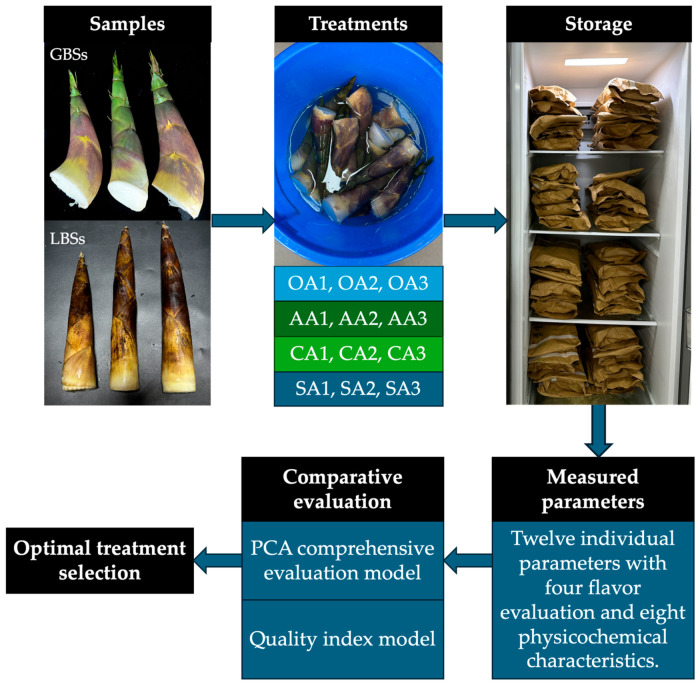
Schematic diagram of this study. GBSs, green bamboo shoots; LBSs, lei bamboo shoots. OA1, OA2 and OA3 represent samples treated with oxalic acid at 2.5 mmol·L^−1^, 5 mmol·L^−1^ and 7.5 mmol·L^−1^, respectively; AA1, AA2 and AA3 represent samples treated with ascorbic acid at 28.4 mmol·L^−1^, 56.8 mmol·L^−1^, and 85.2 mmol·L^−1^, respectively; CA1, CA2 and CA3 represent samples treated with critic acid at 26 mmol·L^−1^, 52 mmol·L^−1^ and 78 mmol·L^−1^, respectively; SA1, SA2 and SA3 represent samples treated with salicylic acid at 0.5 mmol·L^−1^, 1 mmol·L^−1^ and 1.5 mmol·L^−1^, respectively. PCA, principal component analysis.

LBSs and GBSs were harvested from local farms in Lin’an (Zhejiang, China) and Fu’an (Fujian, China) districts, respectively. Uniform LBSs (basal diameter about 4.5 cm and length about 25 cm) and GBSs (basal diameter about 9 cm and length about 20 cm) free of visible wounding and defects were selected, covered with shade bags, put into insulated boxes with ice bags, and then transported to the laboratory on the day of harvest. Before each treatment, dirt and impurities were removed from the shoot surface, and the aged parts were cut off at the bases. Then, the shoots were immersed in the following different solutions (the concentrations were chosen based on a previous study on postharvest application of these acids to vegetables and fruits; three levels (low, medium, high) around the effective concentrations reported in earlier studies were selected) for ten minutes: oxalic acid at 2.5 mmol·L^−1^ (OA1), 5 mmol·L^−1^ (OA2) and 7.5 mmol·L^−1^ (OA3); ascorbic acid at 28.4 mmol·L^−1^ (AA1), 56.8 mmol·L^−1^ (AA2) and 85.2 mmol·L^−1^ (AA3); critic acid at 26 mmol·L^−1^ (CA1), 52 mmol·L^−1^ (CA2) and 78 mmol·L^−1^ (CA3); and salicylic acid at 0.5 mmol·L^−1^ (SA1), 1 mmol·L^−1^ (SA2) and 1.5 mmol·L^−1^ (SA3). After immersion, the bamboo shoots were drained on a rack until dry. Bamboo shoots immersed in distilled water were set as the control (CK). Shaded bags with two ventilation holes made of wood pulp were used to pack the bamboo shoot samples, and then the samples were stored in a refrigerator at 4 °C. The bamboo shoots were sampled on the 3rd, 7th, 10th and 14th days. A road map of the study design is shown in Figure 1.

### 2.2. Measurement of Browning Degree and Mold Degree

According to the method of Zheng et al. [24], browning degree evaluation can directly reflect the surface appearance that consumers perceive. Browning degree was expressed by calculating the percentage of the bottom browning area to the total bottom area. The classification standard is as follows: 1, no browning; 2, browned area < 20%; 3, 20% < browned area < 50%; and 4, browned area > 50%. By observing and counting the macromolds growing on the surface of the bamboo shoot sheaths, the mold degree was expressed as the percentage of the area where the molds grew to the surface area of the bamboo shoot sheaths. The graded standard is as follows: 1, no moldiness; 2, moldiness area < 20%; 3, 20% < moldiness area < 50%; and 4, moldiness area > 50%.

### 2.3. Measurement of Soluble Protein and Soluble Sugar Contents

The soluble protein content in the bamboo shoots was measured by the Coomassie brilliant blue G-250 method [30]. One gram of fresh bamboo shoots was homogenized in 2 mL of distilled water and then centrifuged at 12,000× *g* for 15 min at 4 °C. Subsequently, 1 mL of the clear supernatant was mixed with 5 mL of Coomassie brilliant blue G-250 (Macklin, Shanghai, China) reagent. After a 5 min incubation at 4 °C, the absorbance was measured spectrophotometrically at 595 nm. The soluble protein concentration was quantified based on a standard curve prepared with bovine serum albumin (BSA).

The soluble sugar content in the bamboo shoots was measured by the anthrone method [31]. One gram of dried bamboo shoots was extracted with 15 mL of distilled water in a boiling water bath for 20 min. After cooling, the extract was filtered to obtain a clear solution for analysis. For the colorimetric assay, 0.5 mL of anthrone reagent (dissolved in ethyl acetate) and 5 mL of concentrated sulfuric acid were added to 2 mL of the filtered extract. And then, the mixture was heated in boiling water for 10 min. The absorbance of the solution was measured at 630 nm. The soluble sugar concentration in the samples was calculated based on a standard curve prepared with known concentrations of glucose.

### 2.4. Measurement of Weight Loss

Weight loss was calculated as the percentage reduction from the initial weight [32].

(1) $$Weight\;loss\left(\%\right)=\frac{M_{0}-M_{t}}{M_{0}}\times 100\%$$

where M_0_ is the initial weight and M_t_ is the sample weight at the sampling time point.

### 2.5. Measurement of Lignin and Cellulose Contents

Lignin was extracted and measured according to the method of Morrison et al. [33]. One gram of dried bamboo shoots was homogenized in 5.0 mL of pre-cooled 95% ethanol, and the solution was then centrifuged in a refrigerated centrifuge at 4 °C at 4500× *g* for 7 min. The resultant pellet was sequentially washed three times with 5 mL of 95% ethanol, followed by three washes with a 1:2 (*v*/*v*) ethanol/n-hexane mixture. Acetylation was performed by adding 5 mL of a freshly prepared 25% (*v*/*v*) acetyl bromide in glacial acetic acid solution. The mixture was incubated in a water bath at 70 °C for 30 min, and the reaction was terminated by adding 0.9 mL of 2 M sodium hydroxide solution. After centrifugation at 4500× *g* for 7 min, the absorbance of the supernatant was measured at 280 nm. The lignin content was expressed as the absorbance at 280 nm per gram of dry shoot material.

Cellulose was extracted and measured according to the method of Chen et al. [34]. Ten grams of a frozen sample was homogenized and extracted in 50 mL of Tris-HCl buffer (50 mmol/L, pH 7.2) containing 35 mmol/L sodium dodecyl sulfate for 3 h at room temperature; then, the suspension was centrifuged at 14,000× *g* for 15 min. The resulting pellet was sequentially washed with distilled water, ethanol, and acetone. Fifty milligrams of the dried residue was incubated in 5 mL of 2 mol/L trifluoroacetic acid at 120 °C for 90 min, and then the remaining cellulose was centrifuged and washed with water and ethanol. The obtained pellet was solubilized in 5 mL of 12 mol/L H_2_SO_4_ at 37 °C for 60 min. Finally, the hydrolysate was mixed with fresh anthrone reagent (0.2% in concentrated H_2_SO_4_), heated in a boiling water bath for 5 min, and cooled to room temperature. The cellulose content was calculated according to the absorbance measured at 620 nm.

The contents of lignin and cellulose were expressed as a percentage of fresh weight.

### 2.6. Measurement of Sweetness, Bitterness, Astringency and Umami

The taste scores of GBSs and LBSs, including sweetness, bitterness, astringency and umami, were analyzed using an electronic tongue (YingSheng, Beijing, China) according to the method of Cai et al. [35]. Briefly, ten grams of bamboo shoot samples was ground in a mortar and transferred to a special detection cup for the electronic tongue. And then, 80 mL of distilled water was added and mixed evenly. The number of measurement cycles was set to four. During data processing, the first cycle was discarded, and the average data from the last three cycles were taken as the test results.

### 2.7. PAL, POD and PPO Activity Measurement

PAL activity was determined following the method described by Chen et al. [36]. Briefly, three grams of a sample was homogenized with 10 mL of 100 mmol·L^−1^ boric acid–borax extraction buffer (containing 40 g polyvinylpolypyrrolidone, 35 µL β-mercaptoethanol, and 2 mmol EDTA per liter) in an ice bath. The homogenate was centrifuged at 10,000 r·min^−1^ for 30 min at 4 °C. Subsequently, 1 mL of the supernatant was mixed with 6 mL of 50 mmol·L^−1^ boric acid–borax buffer and 1.0 mL of 20 mmol·L^−1^ L-phenylalanine. The absorbance at 290 nm was measured immediately after mixing. Following incubation at 37 °C in a water bath for 1 h, the absorbance at 290 nm was recorded again.

POD activity was measured according to Liu et al.’s method [37]. Specifically, three grams of a sample was accurately weighed, homogenized, and resuspended in 10 mL of acetate extraction buffer (containing 40 g·L^−1^ PVPP, 1% Triton X-100, and 1 mmol·L^−1^ PEG). The homogenate was then centrifuged at 10,000 rpm for 30 min at 4 °C. Subsequently, 1 mL of the supernatant was mixed with 6 mL of 25 mmol·L^−1^ guaiacol solution and 400 µL of 0.5 mol·L^−1^ H_2_O_2_. The absorbance at 470 nm was measured immediately after mixing.

PPO activity was assayed using the method of Yang et al. [38]. Briefly, ten milligrams of a sample was homogenized in 0.05 mmol·L^−1^ sodium phosphate buffer. The homogenate was subsequently dissolved in 0.1 mmol·L^−1^ sodium phosphate buffer, incubated for 10 min, and centrifuged at 4 °C for 30 min. The resulting supernatant (3 mL) was then mixed with 3.9 mL of sodium phosphate buffer and 1 mL of catechol solution, followed by incubation in a 37 °C water bath for 10 min. Thereafter, 2 mL of 20% trichloroacetic acid (TCA) solution was added to terminate the reaction. Absorbance was measured at 420 nm using 0.05 mmol·L^−1^ sodium phosphate buffer as a blank in place of the enzyme solution.

The activities of PAL, POD and PPO were expressed in units per gram per minute (U·g^−1^·min^−1^).

### 2.8. Principal Component Analysis (PCA) Comprehensive Evaluation

PCA was initially conducted to reduce data dimensionality and to identify the critical variables responsible for distinguishing among samples. The suitability of the dataset for PCA was assessed using Bartlett’s test of sphericity and the Kaiser–Meyer–Olkin (KMO) measure of sampling adequacy. Although not strictly obligatory, the KMO test is widely recognized as a diagnostic tool for evaluating the extent to which variance in the data may arise from underlying common factors. A high KMO value suggests that PCA can yield well-defined and reliable components, thereby reinforcing the validity of the dimensionality reduction process. Consequently, performing this test served as a critical preliminary step in confirming the appropriateness of PCA for the dataset. The number of principal components (PCs) retained was determined based on Kaiser’s eigenvalue criterion (eigenvalue > 1) and Cattell’s scree plot. From the PCA output, factor scores were extracted for each treatment, enabling the evaluation of spatial distributions among samples in terms of preservation quality. In addition, factor loadings were analyzed to interpret the relative contribution of each original variable to the extracted components [25]. The function expressions of the principal component score (PCS) of GBSs (PCSG) and LBSs (PCSL) were obtained by dividing the component coefficient by the arithmetic square root of the eigenvalue corresponding to the principal component [26], as shown in Equations (2) and (3). The PCA comprehensive evaluation model was obtained by dividing the product of each principal component score and the corresponding variance contribution rate by the sum of the variance contribution rates [26], as expressed in Equations (4) and (5).

(2) $$\begin{array}{c} PCSG=-0.027X_{1}-0.027X_{2}+0.028X_{3}+0.028X_{4}+0.028X_{5}-0.027X_{6}\\ \;\;\;\;\;\;\;\;\;\;\;\;\;\;\;\;\;\;\;\;\;\;\;\;+0.026X_{7}+0.027X_{8}-0.026X_{9}+0.028X_{10}+0.028X_{11}+0.027X_{12} \end{array}$$

(3) $$\begin{array}{c} PCSL=-0.028X_{1}-0.027X_{2}+0.028X_{3}+0.029X_{4}+0.030X_{5}-0.028X_{6}\\ \;\;\;\;\;\;\;\;\;\;\;\;\;\;\;\;\;\;\;\;\;\;\;\;+0.028X_{7}+0.027X_{8}-0.028X_{9}+0.028X_{10}+0.030X_{11}+0.027X_{12} \end{array}$$

(4) Comprehensive score (GBS)=PCSG

(5) Comprehensive score (LBS)=PCSL

where X_1_ through X_12_ denote soluble protein and soluble sugar contents; weight loss; lignin and cellulose contents; relative sweetness, bitterness, astringency and umami values; and activities of PAL, POD, and PPO, respectively.

### 2.9. Quality Index (Q_i_)

To quantitatively evaluate the overall quality of bamboo shoot samples throughout storage, a composite quality index (Q_i_), which ranges from 0 to 1, was established based on the integrated assessment of sensory attributes and physicochemical properties, serving as a metric for normalizing and expressing variables relative to the minimum value of the control parameter. The Q_i_ comprises 12 individual parameters, with four flavor evaluation (sweetness, bitterness, astringency and umami) and eight physicochemical (soluble protein, soluble sugar, weight loss, lignin, cellulose, PAL, POD, and PPO) characteristics, thereby enabling a comprehensive measure of product quality over time. A Q_i_ value of 1.0 corresponds to optimal quality, whereas values approaching 0.0 indicate progressive deterioration [27]. To achieve parameter standardization, the subsequent formula can be employed:

(6) Xi^=Xi−XminXmax−Xmin

where Xi^ represents the normalized value of the quality parameter *X*, while $X_{i}$ stands for the value of the quality parameter measured. $X_{max}$ and $X_{min}$ denote the maximum and minimum values of the quality parameter X across the entire dataset, respectively. The Q_i_ was computed as follows:

(7) Qi=∑i=1NXi^N

with ‘*N*’ representing the number of parameters. The generated Q_i_ accommodated both normalized objective sample properties and the overall sensory data.

### 2.10. Statistical Analysis

All data were expressed as mean ± SD (*n* = 3). One-way analysis of variance (ANOVA), PCA and bivariate correlations with SPSS 17.0 statistical software (SPSS Inc., Chicago, IL, USA) were applied for statistical analysis. Significant differences were calculated according to Tukey’s multiple range tests. Differences at *p* < 0.05 (*) were statistically significant. The Pearson correlation coefficient (r) was used to indicate bivariate correlations: a *p*-value > 0.05 indicated no correlation, 0.01 < *p* < 0.05 indicated correlation, and *p* < 0.01 indicated significant correlation.

## 3. Results and Discussion

### 3.1. Effect on Appearance Quality of Two Bamboo Shoot Species

**Figure 2:**
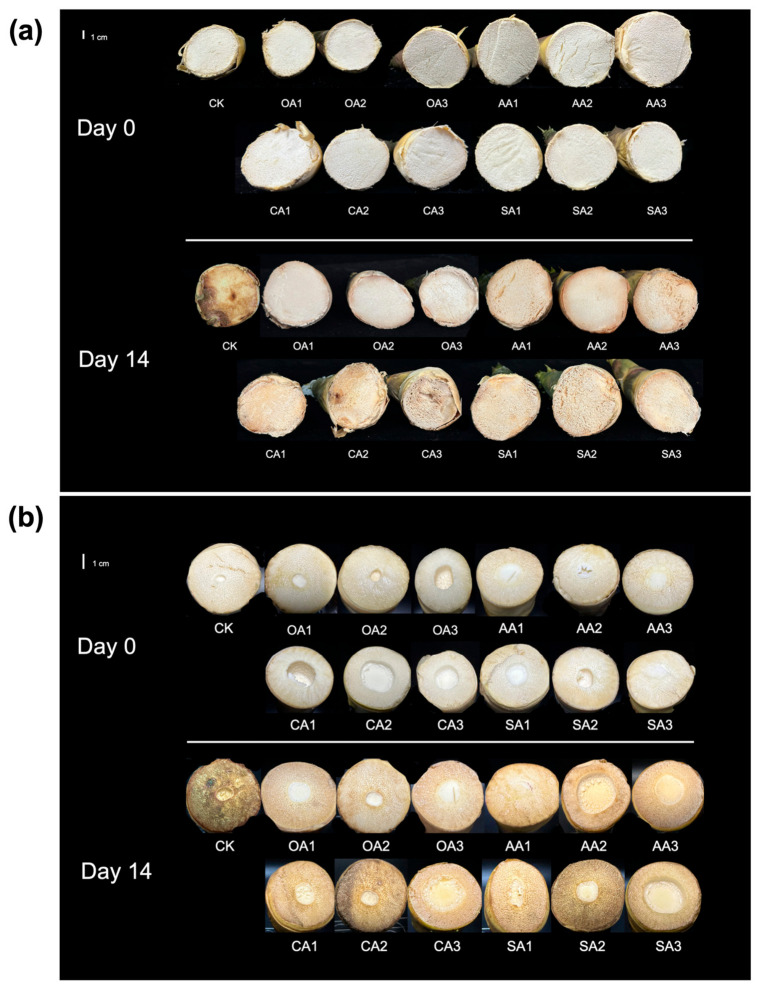
Effect of different organic acid treatments on basal appearance of (**a**) GBSs and (**b**) LBSs on day 3 and day 14. GBSs, green bamboo shoots; LBSs, lei bamboo shoots. CK represents control samples; OA1, OA2 and OA3 represent samples treated with oxalic acid at 2.5 mmol·L^−1^, 5 mmol·L^−1^ and 7.5 mmol·L^−1^, respectively; AA1, AA2 and AA3 represent samples treated with ascorbic acid at 28.4 mmol·L^−1^, 56.8 mmol·L^−1^, and 85.2 mmol·L^−1^, respectively; CA1, CA2 and CA3 represent samples treated with critic acid at 26 mmol·L^−1^, 52 mmol·L^−1^ and 78 mmol·L^−1^, respectively; SA1, SA2 and SA3 represent samples treated with salicylic acid at 0.5 mmol·L^−1^, 1 mmol·L^−1^ and 1.5 mmol·L^−1^, respectively.

**Figure 3:**
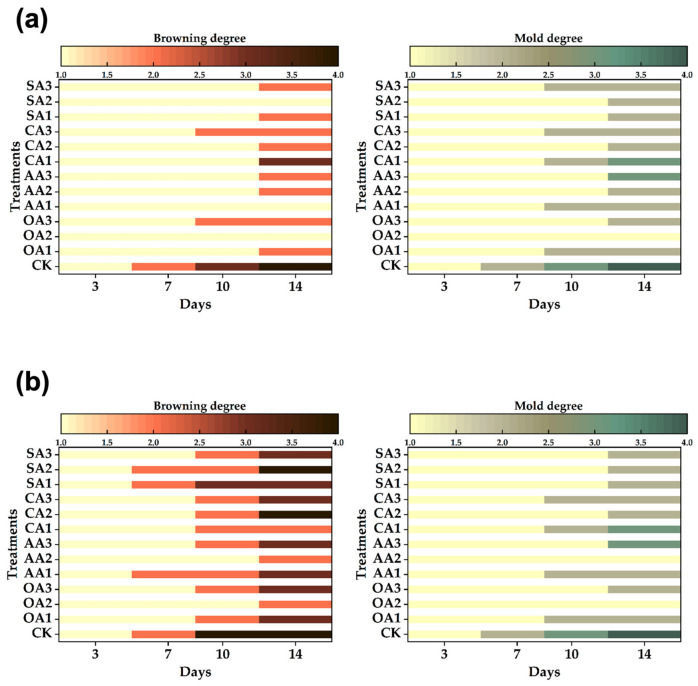
Effect of different organic acid treatments on browning degree and mold degree of (**a**) GBSs and (**b**) LBSs: 1, no browning or moldiness; 2, browned area < 20% and moldiness area < 20%; 3, 20% < browned area < 50% and 20% < moldiness area < 50%; and 4, browned area > 50% and moldiness area > 50%. GBSs, green bamboo shoots; LBSs, lei bamboo shoots. CK represents control samples; OA1, OA2 and OA3 represent samples treated with oxalic acid at 2.5 mmol·L^−1^, 5 mmol·L^−1^ and 7.5 mmol·L^−1^, respectively; AA1, AA2 and AA3 represent samples treated with ascorbic acid at 28.4 mmol·L^−1^, 56.8 mmol·L^−1^, and 85.2 mmol·L^−1^, respectively; CA1, CA2 and CA3 represent samples treated with critic acid at 26 mmol·L^−1^, 52 mmol·L^−1^ and 78 mmol·L^−1^, respectively; SA1, SA2 and SA3 represent samples treated with salicylic acid at 0.5 mmol·L^−1^, 1 mmol·L^−1^ and 1.5 mmol·L^−1^, respectively.

The basal appearance of the CK and of two species of bamboo shoots treated with different organic acids after storage at 4 °C for 0 and 14 days is presented in Figure 2. By visual observation during storage, treatments with various organic acids inhibited basal browning and mildew development on the sheath in both GBSs (Figure 3a) and LBSs (Figure 3b). For GBSs, after 7 days of storage, the surface of the CK exhibited mild browning and moldiness, whereas no browning and moldiness were observed in any of the organic acid-treated shoots. By day 14, browning and moldiness were more severe in the CK, and OA2 best preserved the appearance quality of GBSs, as there was no browning and moldiness on the surface of OA2-treated shoots. This result aligns with the finding of Cheng et al. [39], who reported that oxalic acid suppressed enzymatic browning in fresh-cut yam by modulating gene expression related to the phenylpropanoid pathway and browning-related enzymes. And it was shown that oxalic acid could decrease macroscopic fungal growth or bacterial lesions on the surface of bamboo shoots [24]. For LBSs, at day 10, severe browning and moderate moldiness occurred on the surface of the CK, which was notably more pronounced than in organic acid-treated shoots. In contrast, SA3 most effectively maintained the appearance quality of LBSs during storage. This result is consistent with that of Zhang et al. [40], who demonstrated that salicylic acid delayed pericarp browning in fresh longans by enhancing reactive oxygen species (ROS) scavenging capacity. Thus, the OA2 and SA3 treatments may induce gene expression related to antioxidant enzymes or ROS scavenging capacity, resulting in delayed bottom browning. In summary, our results confirm the efficacy of oxalic acid (5 mmol·L^−1^) and salicylic acid (1.5 mmol·L^−1^) in retarding the decline in appearance quality of GBSs and LBSs, respectively, during storage.

### 3.2. Effect on the Soluble Protein and Soluble Sugar Contents of Two Bamboo Shoots Species

As shown in Table 1, the soluble protein contents of the CK and shoots treated with different organic acids decreased during the storage period. For GBSs, the soluble protein content of the CK decreased faster than that of the organic acid treatments, and the SA1 treatment demonstrated the most effective inhibition of soluble protein loss. Similarly, in LBS samples treated with AA2, AA3, CA2, CA3, SA1, and SA2, the soluble protein content was maintained at higher levels compared to the CK after 14 days of storage. Among these, the AA2 treatment most effectively suppressed the decline in soluble protein content in LBSs. The soluble sugar contents in both GBSs and LBSs exhibited a trend similar to that of soluble protein (Table 2). However, the SA2 treatment was the most effective in mitigating the reduction in soluble sugar contents in both GBSs and LBSs during storage. These results are consistent with those in Nazari et al.’s [41] report on Bidane Sefid at the unripening stage, which found that salicylic acid at 0.1 mM was associated with increased soluble sugar in the leaves and berry skin, and salicylic acid at 1 mM significantly increased the total protein of the berry flesh and leaves.

Soluble sugar and soluble protein are key nutritional components of bamboo shoots, serving as important indicators for evaluating vegetable quality and nutritional value, and are involved in various metabolic processes within shoots [42]. All biological activities in bamboo shoots require substantial energy, which is primarily derived from the degradation of non-structural carbohydrates (NSCs), including starch and soluble sugars, as well as proteins and amino acids (PAs) [43]. Lu and Xu [44] also reported that the hydrolysis of carbohydrates leads to an increase in total soluble sugar content, whereas these sugars are concurrently reduced due to respiratory consumption in bamboo shoots. According to previous studies, oxalic acid or gamma radiation could inhibit the decrease in total sugar by suppressing respiration in bamboo shoots [3,24]; thus, the AA2, SA1 and SA2 treatments may control the decrease in soluble protein and sugar by restraining respiration in GBSs and LBSs. In general, this study has proved that ascorbic acid (56.8 mmol·L^−1^) and salicylic acid (0.5 mmol·L^−1^) play a positive role in retarding the decline in soluble protein content in GBSs and LBSs, respectively. Furthermore, postharvest treatment with salicylic acid at 1 mmol·L^−1^ maintains the most stable soluble sugar content in both GBSs and LBSs throughout the storage period.

### 3.3. Effect on Weight Loss of Two Bamboo Shoots Species

As shown in Table 3, the pattern of weight loss change was similar in the CK and shoots treated with different organic acids during storage, with all groups exhibiting a gradual increase in weight loss over time, which may be partly caused by the decrease in soluble protein and soluble sugar. The weight loss of fresh fruits and vegetables is mainly due to water loss caused by respiration and transpiration [45]. Notably, compared with the CK, the OA2 and AA2 treatments most effectively reduced weight loss in GBSs and LBSs, respectively. These treatments may alleviate the weight reduction of bamboo shoots by inhibiting their respiration and transpiration. These results are consistent with previous studies; for example, it was shown that oxalic acid can mitigate the increase in cut-end dehydration of fresh-cut green and purple asparagus effectively [46]; and Zarbakhsh et al. [47] reported that ascorbic acid inhibited water loss in processed arils better than a control. This study suggests that oxalic acid at 5 mmol·L^−1^ and ascorbic acid at 56.8 mmol·L^−1^ exert beneficial effects in mitigating the increase in weight loss for GBSs and LBSs, respectively.

### 3.4. Effect on Lignin and Cellulose Contents of Two Bamboo Shoot Species

The contents of lignin and cellulose gradually increased in both the CK and the various organic acid-treated shoots during storage (Table 4 and Table 5). The primary cell wall is primarily composed of pectin, cellulose, and hemicellulose, which together constitute the matrix that determines its mechanical properties [48]. Alterations in the activities of pectinase, cellulase, and glycosidase may influence the pectin matrix, polysaccharide chains, and the degree of esterification of polygalacturonic acid chains within the cell wall [48,49]. Lignin biosynthesis constitutes a complex phenylpropanoid metabolic pathway involving the synergistic action of multiple enzymes, including PAL, cinnamate 4-hydroxylase (C4H), 4-coumarate:CoA ligase (4CL), CAD, and POD. During monolignol biosynthesis, PAL first catalyzes the deamination of L-phenylalanine to produce trans-cinnamic acid, which is subsequently converted into various phenylpropanoid compounds, such as monolignols, flavonoids, and chlorogenic acid. Among these enzymes, CAD catalyzes the final step in monolignol biosynthesis and plays a crucial role in determining lignin diversity [50]. C4H and 4CL act at the branch points of the phenylpropanoid biosynthetic pathway, linking lignin biosynthesis to the flavonoid branch pathway by catalyzing the conversion of hydroxycinnamic acids to their corresponding coenzyme A esters [50]. POD catalyzes the polymerization of lignin precursors, thereby completing the final stage of lignin formation [51]. The lignin and cellulose contents were significantly lower in all organic acid treatments compared with those of the CK throughout storage. Among these treatments, GBSs treated with AA1 and LBSs treated with AA2 exhibited the most pronounced effect in retarding the increase in lignin content (Table 4). Similarly, the retardation of cellulose content increase was most effective in GBSs treated with OA1 and in LBSs treated with AA2 (Table 5). Zheng et al. [24] reported that 10 mM oxalic acid treatment significantly delayed the accumulation of lignin in bamboo shoots during cold storage and inhibited the activities of PAL, CAD, 4CL, and POD. A previous study found thickened cell walls and decompartmentalization of cellular structure in exogenous H_2_O_2_-treated shoots, demonstrating that endogenous H_2_O_2_ may play a vital role in the lignification process of bamboo shoots [52]. Treatments with AA1, AA2 and OA1 may change lignin and cellulose contents by modifying related enzyme activities and endogenous H_2_O_2_ content. Therefore, through this study, it was concluded that oxalic acid at 2.5 mmol·L^−1^ and ascorbic acid at 28.4 mmol·L^−1^ and 56.8 mmol·L^−1^ had positive effects on suppressing the accumulation of lignin and cellulose.

### 3.5. Effect on Sweetness, Bitterness, Astringency and Umami of Two Bamboo Shoot Species

**Figure 4:**
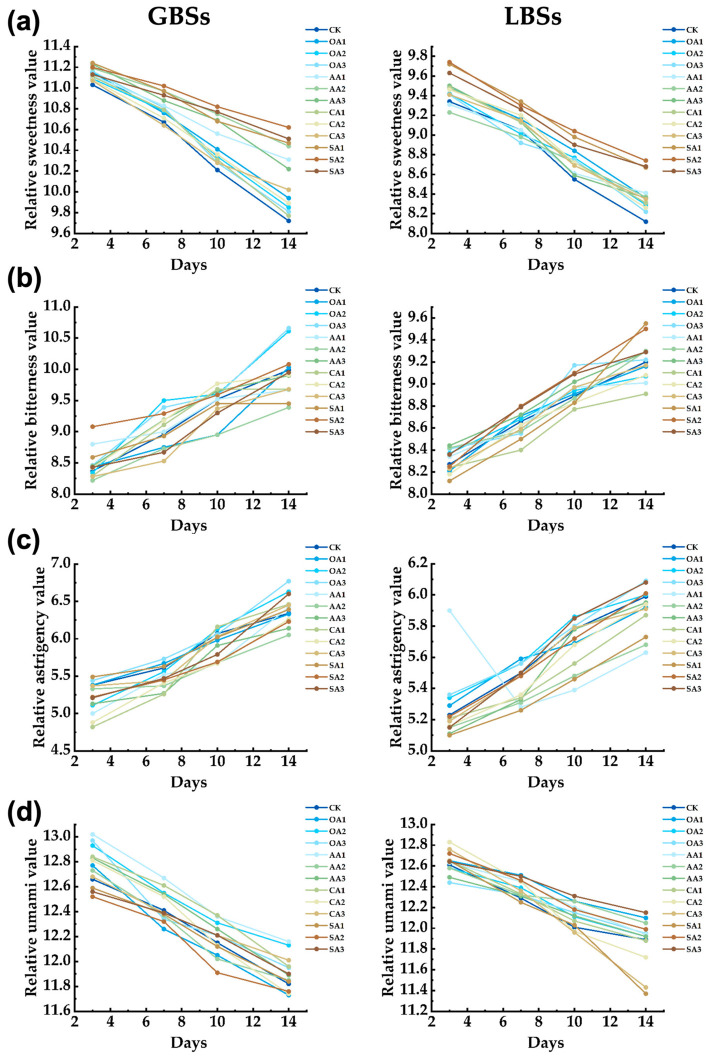
Effect of different organic acid treatments on relative (**a**) sweetness, (**b**) bitterness, (**c**) astringency and (**d**) umami values of GBSs and LBSs. GBSs, green bamboo shoots; LBSs, lei bamboo shoots. CK represents control samples; OA1, OA2 and OA3 represent samples treated with oxalic acid at 2.5 mmol·L^−1^, 5 mmol·L^−1^ and 7.5 mmol·L^−1^, respectively; AA1, AA2 and AA3 represent samples treated with ascorbic acid at 28.4 mmol·L^−1^, 56.8 mmol·L^−1^, and 85.2 mmol·L^−1^, respectively; CA1, CA2 and CA3 represent samples treated with critic acid at 26 mmol·L^−1^, 52 mmol·L^−1^ and 78 mmol·L^−1^, respectively; SA1, SA2 and SA3 represent samples treated with salicylic acid at 0.5 mmol·L^−1^, 1 mmol·L^−1^ and 1.5 mmol·L^−1^, respectively.

The relative sweetness value gradually declined in both the CK and the organic acid-treated shoots during storage (Figure 4a), and all treatments had positive effects on delaying the decrease in sweetness compared to the CK, among which the SA2 treatment was the most effective in both GBSs and LBSs. No significant differences were detected in the relative bitterness (Figure 4b), astringency (Figure 4c), or umami (Figure 4d) values between the different organic acid-treated shoots and the CK. In addition, over the storage period, the relative bitterness and astringency values increased gradually, whereas the relative umami values decreased. Obviously, the AA2 treatment most effectively inhibited the increase in bitterness and astringency in GBSs during storage, while the AA1 treatment best prevented the decrease in umami in GBSs. For LBSs, the CA1 treatment was most effective in retarding the increase in bitterness, and the AA1 treatment most effectively slowed the increase in astringency. In addition, the SA3 treatment best mitigated the decline in umami in LBSs.

Wang et al. [53] found that during the storage of bamboo shoots, the three primary nutrients—proteins, carbohydrates, and lipids—are initially degraded into amino acids, sugars, and fatty acids, which are subsequently converted into various flavor compounds through distinct metabolic pathways. For instance, L-glutathione, produced from protein degradation, can inhibit the generation of off-flavors and prevent flavor loss caused by oxidation during storage [54]. Following carbohydrate degradation, a portion of the resulting sugars serves as precursors for the biosynthesis of L-phenylalanine, which is then metabolized via the phenylalanine pathway, involving enzymes such as PAL, C4H, 4CL, and POD, ultimately leading to lignin synthesis [55]. Another portion of sugars enters the Embden–Meyerhof–Parnas (EMP) pathway to generate pyruvate, which can be further converted into acetyl-CoA, thereby giving rise to various key flavor compounds [53]. N-acetyl-L-glutamine, tyrosine, and oxalic acid derived from the glyoxylate and dicarboxylate metabolic pathways, as well as succinic acid, citric acid, and malic acid produced through the tricarboxylic acid (TCA) cycle, are all major components contributing to the flavor profile of bamboo shoots [53]. The sweetness of bamboo shoots is primarily attributed to their soluble sugar content. During the 14-day storage, the treatments with various organic acids delayed the reduction in soluble sugars and sweetness to different extents compared with the CK, among which the SA treatment exhibited the most pronounced inhibitory effect. Previous studies have shown that *Lactiplantibacillus pentosus* can increase the contents of compounds such as methyl toluene and acetic acid by regulating multiple metabolic pathways, including glycolysis and tyrosine metabolism, thereby contributing to a more intense sour and pungent flavor in fermented bamboo shoots [56]. Therefore, the ability of the SA2 treatment to suppress the reduction in soluble sugar content and simultaneously preserve sweetness suggests that this treatment may primarily act on the regulation of carbohydrate metabolism. By reducing the metabolic flux of sugars toward the phenylpropanoid pathway and the EMP pathway, the SA2 treatment enables a greater proportion of soluble sugars to be retained, thereby preserving the characteristic sweetness of bamboo shoots. Notably, although the SA1 and AA2 treatments effectively mitigated the decrease in soluble protein content of GBSs and LBSs, respectively, none of the treatments exhibited a significant effect on umami compared to the CK, which is due to the fact that umami taste is primarily derived from free amino acids, particularly glutamic acid and aspartic acid, rather than from intact protein. Treatments that suppress protein degradation may inadvertently limit the generation of free amino acids from proteolysis, thereby failing to enhance umami perception and suggesting that maintaining soluble protein content is not equal to preserving umami. Bitterness and astringency in bamboo shoots are primarily attributed to secondary metabolites, including flavonoids, phenolic acids, alkaloids, and tannins, which are synthesized through the phenylpropanoid pathway [57]. A study conducted by Gao et al. [58] also reported that phenylalanine and tryptophan are the key factors contributing to the bitterness of bamboo shoots. The accumulation of these compounds is tightly regulated by transcription factors such as AP2/ERF, MYB, and bHLH families [57]. It was shown that cold plasma treatment suppresses the accumulation of precursors and intermediates involved in lignin metabolism, such as phenylalanine, cinnamic acid, and p-coumaric acid, thereby contributing to the reduction in bitterness in bamboo shoots [59]. In this study, like umami, no treatments had obvious effects on delaying the increase in bitterness and astringency compared to the CK, which may be because these treatments did not sufficiently modulate the biosynthetic pathways responsible for bitter and astringent compounds.

Liu et al. [59] found that after cold plasma treatment, the compounds responsible for unpleasant odors in fresh-cut bamboo shoots, such as 4-hydroxybenzaldehyde (associated with grilled meat and bitter almond notes), were significantly reduced, while green and fruity aromas, such as 2,6-nonadienal and 4-nitrophenol, were effectively preserved. As a result, treated bamboo shoots exhibited a pronounced sweet and oily odor profile. Hence, different organic acid treatments may also affect the contents of flavor compounds by adjusting various metabolic pathways, thus maintaining the sweetness of bamboo shoots. Our results indicated that salicylic acid plays a beneficial role in mitigating the reduction in sweetness of both GBSs and LBSs, as well as in delaying the decrease in umami intensity in LBSs. Additionally, ascorbic acid and citric acid exhibit positive effects by suppressing the increase in bitterness in GBSs and astringency in LBSs, respectively. Furthermore, ascorbic acid also contributes to inhibiting the decline in umami perception in GBSs.

### 3.6. Effect on PAL, POD and PPO Activities of Two Bamboo Shoot Species

During the storage period, PAL activity in GBSs treated with various organic acids gradually increased and remained consistently lower than that in the CK (Table 6). Among these treatments, OA2 resulted in the smallest increase in PAL activity for GBSs over the 14-day storage. In contrast, PAL activity in LBSs followed a different pattern: under the OA3, CA1, CA2, CA3, and CK treatments, it increased during the first 10 days and subsequently declined. By day 14, only CA2-treated LBSs exhibited PAL activity lower than that of the CK. For all organic acid treatments, POD activity in both GBSs and LBSs gradually increased and was consistently and significantly lower (*p* < 0.05) than that in the CK (Table 7). Notably, POD activity of OA2-treated GBSs and OA3-treated LBSs rose the least throughout the 14-day storage.

PAL and POD are key enzymes involved in the phenylpropanoid metabolic pathway. PAL catalyzes the conversion of L-phenylalanine to cinnamic acid, which is subsequently transformed into various phenylpropanoid compounds, including monolignols, flavonoids, and chlorogenic acid [55]. POD catalyzes the polymerization of lignin precursors, representing the final step in lignin formation [51]. In this study, although the OA2 treatment most effectively inhibited the increase in PAL and POD activities in GBSs, and the CA2 and OA3 treatments most effectively suppressed the increase in these enzyme activities in LBSs, these two treatments were less effective than the AA1 and AA2 treatments in reducing lignin accumulation. Although PAL and POD are key enzymes in the lignin biosynthesis pathway, the inconsistency between their activity levels and the final lignin accumulation suggests that the regulation of lignification in bamboo shoots occurs at multiple levels beyond enzyme activity modulation. The activity of these enzymes, measured in vitro under optimal conditions, represents potential catalytic capacity rather than actual in vivo metabolic flux. Therefore, a treatment that effectively suppresses enzyme activity at specific time points does not necessarily translate to the lowest total lignin deposition over the entire storage period. A previous study reported similar findings: although a gamma irradiation treatment at a dose of 5 kGy exhibited the strongest inhibitory effects on PAL and POD activities, it did not achieve the greatest suppression of lignin and cellulose synthesis [4]. This observation suggested that enzyme activities may not be the sole factor influencing lignification during storage [4]. Luo et al. [60] reported that ethylene is also involved in the lignification process of bamboo shoots. In addition, the activities of enzymes such as PAL, CAD, 4CL, and POD, along with the expression of their corresponding genes, collectively regulate lignin synthesis and accumulation [61,62,63]. Existing studies have indicated that lignification in bamboo shoots during storage is primarily regulated by the expression of CAD and POD, rather than by PAL and 4CL. Moreover, the regulation of POD expression may occur at the transcriptional level, whereas the expression of genes such as PAL, CAD, and 4CL may be regulated post-transcriptionally [64]. Postharvest lignification in bamboo shoots is governed by a complex transcriptional network involving transcription factor families such as NAC and MYB, which may participate in the lignification process by modulating the expression of multiple genes related to lignin and cellulose synthesis [1]. Therefore, in this study, the treatments with different organic acids may have affected enzyme activities and lignin content by regulating the expression of genes involved in lignin synthesis in bamboo shoots at various levels.

PPO activity in both GBSs and LBSs generally increased during storage (Table 8). However, in AA3-treated GBS samples, a decline was observed after 10 days. A previous study [65] showed that when fruit or vegetable tissue was damaged or stored under adverse conditions during picking or processing, PPO activity would initially increase during storage. This trend in PPO activity was similar to that found by Yeoh and Ali [66] for fresh-cut pineapple. In GBSs, PPO activities under the OA3 treatment increased the least compared with the CK. For LBSs, PPO activities in all acid-treated samples were lower than the CK within the first 10 days but surpassed the CK after 10 days. Among these, OA2-treated LBSs maintained the lowest PPO activity throughout the storage period.

PPO promotes browning during the storage of bamboo shoots by catalyzing the oxidation of phenolic compounds. Additionally, PPO accelerates the lignification process by facilitating the oxidation of phenolic substances such as chlorogenic acid and coumarin [4]. PAL serves as a key enzyme in the biosynthesis of phenolic compounds, and increased PAL activity may lead to elevated levels of polyphenol precursors. Whether metabolic flux is directed toward polyphenol or lignin biosynthesis depends on the activity of downstream enzymes [67]. These observations suggest a competitive relationship between browning and lignification in the consumption of phenolic substrates in bamboo shoots during storage. Moreover, the browning process involves a complex regulatory network encompassing multiple enzymes and gene expression. This complexity explains why the OA2 and OA3 treatments, despite exhibiting greater inhibitory effects on the PPO activities of GBSs and LBSs, were not the most effective in suppressing browning based on visual evaluation. Like lignification, the effects of different organic acid treatments on browning intensity in bamboo shoots may be mediated by the differential regulation of browning-related gene expression at multiple levels. In general, our results confirmed that oxalic acid at 5 mmol·L^−1^ and 7.5 mmol·L^−1^ could effectively inhibit PAL, POD and PPO activities.

### 3.7. Modeling of PCA Comprehensive Evaluation

**Figure 5:**
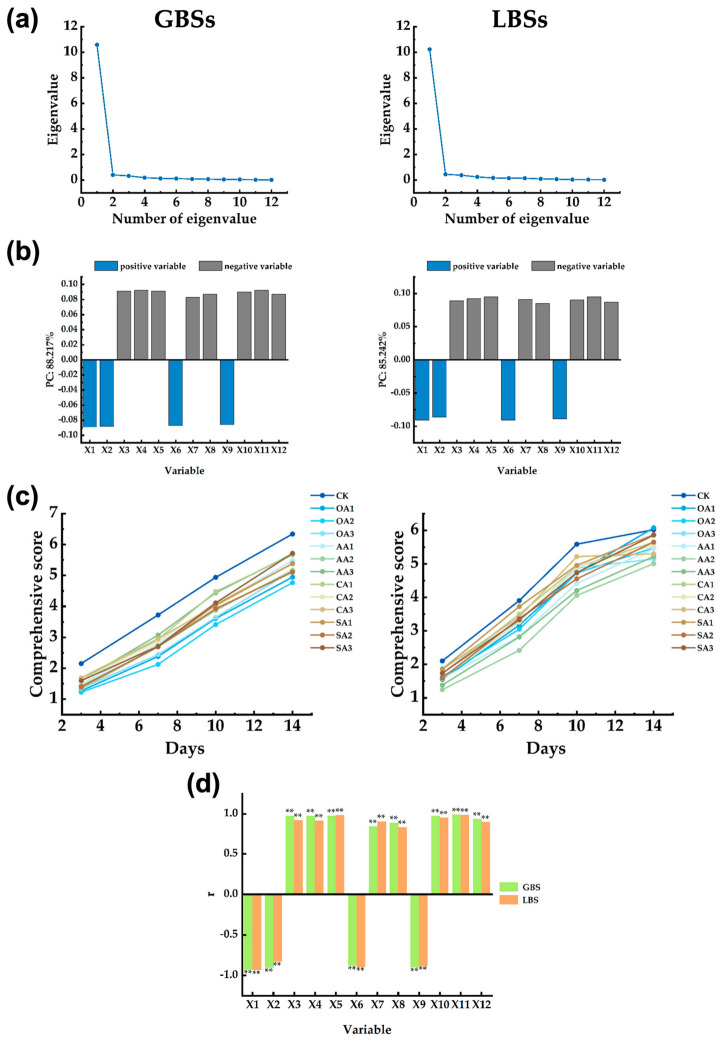
(**a**) Cattel scree plots displaying the estimated number of principal components in PCA. (**b**) Component coefficient of the variables, showing the strength and direction of each bamboo shoot quality parameter contribution to a given principal component determined in PCA. (**c**) Comprehensive scores of bamboo shoot samples treated with different organic acids during storage. (**d**) Correlation analysis (2-tailed) of comprehensive scores and quality variables. GBSs, green bamboo shoots; LBSs, lei bamboo shoots; PC, principal component; X1, soluble protein content; X2, soluble sugar content; X3, weight loss; X4, lignin content; X5, cellulose content; X6, relative sweetness value; X7, relative bitterness value; X8, relative astringency value; X9, relative umami value; X10, activity of PAL; X11, activity of POD; X12, activity of PPO; r, Pearson correlation coefficient; **, *p* < 0.01. CK represents control samples; OA1, OA2 and OA3 represent samples treated with oxalic acid at 2.5 mmol·L^−1^, 5 mmol·L^−1^ and 7.5 mmol·L^−1^, respectively; AA1, AA2 and AA3 represent samples treated with ascorbic acid at 28.4 mmol·L^−1^, 56.8 mmol·L^−1^, and 85.2 mmol·L^−1^, respectively; CA1, CA2 and CA3 represent samples treated with critic acid at 26 mmol·L^−1^, 52 mmol·L^−1^ and 78 mmol·L^−1^, respectively; SA1, SA2 and SA3 represent samples treated with salicylic acid at 0.5 mmol·L^−1^, 1 mmol·L^−1^ and 1.5 mmol·L^−1^, respectively.

Based on PCA of GBSs, a single principal component (PC) with an eigenvalue greater than 1.0 was extracted from the four taste indicators and eight physicochemical parameters (Figure 5a). As illustrated in the PCA loading plot (Figure 5b), this PC accounted for 88.22% of the total variance, indicating that it effectively captured the original flavor and physicochemical characteristics of GBSs. Similarly, PCA of LBSs also yielded one PC with an eigenvalue exceeding 1.0 (Figure 5a), explaining 85.24% of the variance (Figure 5b), thereby sufficiently representing the flavor and physicochemical profile of LBSs. The loading plots (Figure 5b) further revealed that for both GBSs and LBSs, soluble protein and soluble sugar contents and relative sweetness and umami values exhibited strong negative loadings on the extracted PC. In contrast, weight loss, lignin and cellulose contents, relative bitterness and astringency values, and the activities of PAL, POD, and PPO displayed high positive loadings. This distribution suggests that the PC primarily reflects the dynamic trade-off between the deterioration in nutritional and flavor quality and the progression of senescence and lignification in bamboo shoots during postharvest storage. The component coefficient and eigenvalues for the selected PCs (eigenvalue > 1.0) are in Figure 5a,b. As demonstrated in Figure 5d, correlation analysis (two-tailed) revealed that the comprehensive score was significantly negatively correlated with soluble protein and soluble sugar contents, relative sweetness and umami values. Conversely, it was significantly positively correlated with weight loss, lignin and cellulose contents, relative bitterness and astringency values, and activities of PAL, POD, and PPO. These findings further validate that the comprehensive score obtained through the PCA comprehensive evaluation model effectively captures the opposing trends between quality retention and senescence-related deterioration indicators in bamboo shoots during storage.

As shown in Figure 5c, bamboo shoot samples exhibiting high quality corresponded to low comprehensive scores, while the CK consistently maintained the highest comprehensive score throughout the 14-day storage period. This finding suggests that all organic acid treatments contributed to delaying the quality deterioration of bamboo shoots to varying degrees. Notably, samples of GBSs treated with OA2 and LBSs treated with AA2 consistently recorded the lowest comprehensive scores throughout storage. Moreover, the comprehensive score of OA2-treated GBSs remained lower than that of AA2-treated LBSs throughout the entire storage duration, implying that the preservation effectiveness of different organic acids is influenced by both bamboo shoot variety and storage time. These observations align with earlier research indicating that preharvest application of oxalic acid may enhance yield and fruit quality in pomegranates while also increasing the levels of bioactive constituents with health benefits [68]. Similarly, Zou et al. [69] reported that incorporating ascorbic acid into a chitosan-based edible coating, particularly when combined with plasma-activated water treatment, effectively preserved the nutritional and sensory attributes of red grapes and extended their shelf life by a minimum of eight days.

### 3.8. Modeling of the Quality Index (Q_i_)

**Figure 6:**
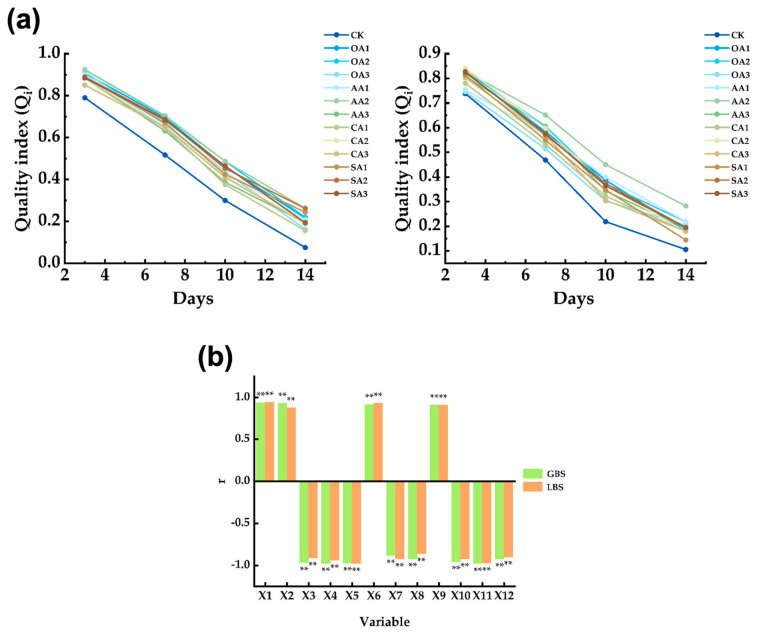
(**a**) Quality index (Q_i_) values of bamboo shoot samples treated with different organic acids during storage. (**b**) Correlation analysis (2-tailed) of Q_i_ values and quality variables. GBSs, green bamboo shoots; LBSs, lei bamboo shoots; X1, soluble protein content; X2, soluble sugar content; X3, weight loss; X4, lignin content; X5, cellulose content; X6, relative sweetness value; X7, relative bitterness value; X8, relative astringency value; X9, relative umami value; X10, PAL activity; X11, POD activity; X12, PPO activity; r, Pearson correlation coefficient; **, *p* < 0.01. CK represents control samples; OA1, OA2 and OA3 represent samples treated with oxalic acid at 2.5 mmol·L^−1^, 5 mmol·L^−1^ and 7.5 mmol·L^−1^, respectively; AA1, AA2 and AA3 represent samples treated with ascorbic acid at 28.4 mmol·L^−1^, 56.8 mmol·L^−1^, and 85.2 mmol·L^−1^, respectively; CA1, CA2 and CA3 represent samples treated with critic acid at 26 mmol·L^−1^, 52 mmol·L^−1^ and 78 mmol·L^−1^, respectively; SA1, SA2 and SA3 represent samples treated with salicylic acid at 0.5 mmol·L^−1^, 1 mmol·L^−1^ and 1.5 mmol·L^−1^, respectively.

As depicted in Figure 6a, a consistent reduction in Q_i_ values was observed throughout the storage period, reflecting a gradual decline in both flavor and physicochemical properties. Throughout the entire storage duration, the CK consistently exhibited the lowest Q_i_ values for both GBSs and LBSs. Notably, samples treated with AA2 maintained the highest Q_i_ values over the 14-day storage, with GBSs showing higher values than LBSs, suggesting that the preservation efficacy of the AA2 treatment may be influenced by bamboo shoot type. These observations are consistent with prior studies that have established ascorbic acid as an effective agent for extending the postharvest shelf life of fruits or vegetables. For instance, Liu et al. [70] reported that exogenous application of ascorbic acid significantly enhanced the quality attributes and storability of harvested longan fruit, thereby prolonging its shelf life. In contrast to the findings derived from the PCA comprehensive evaluation model, correlation analysis (two-tailed) (Figure 6b) demonstrated that the Q_i_ values exhibited a significant positive correlation with soluble protein and soluble sugar contents, relative sweetness and umami values. On the other hand, they were significantly negatively associated with weight loss, lignin and cellulose accumulation, and relative bitterness and astringency values, as well as activities of PAL, POD, and PPO. These results provide further evidence that the Q_i_ value effectively captures the concurrent trends between preservation quality and senescence-associated deterioration indicators in bamboo shoots throughout postharvest storage.

### 3.9. Comparative Analysis of the PCA Comprehensive Evaluation Model and the Q_i_ Model

**Figure 7:**
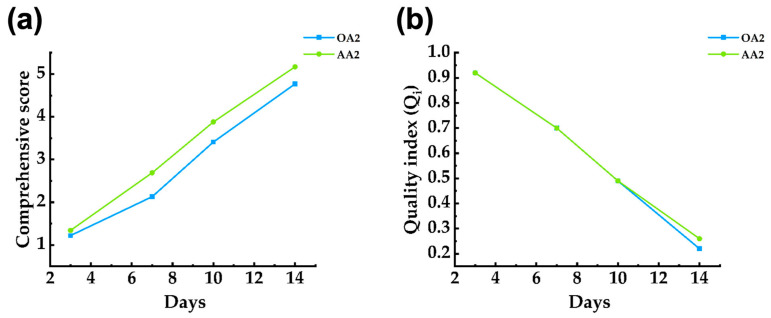
(**a**) Comprehensive scores and (**b**) quality index (Q_i_) values of GBS samples treated with OA2 or AA2 during storage. GBSs, green bamboo shoots. OA2 represents samples treated with oxalic acid at 5 mmol·L^−1^; AA2 represents samples treated with ascorbic acid at 56.8 mmol·L^−1^.

**Figure 8:**
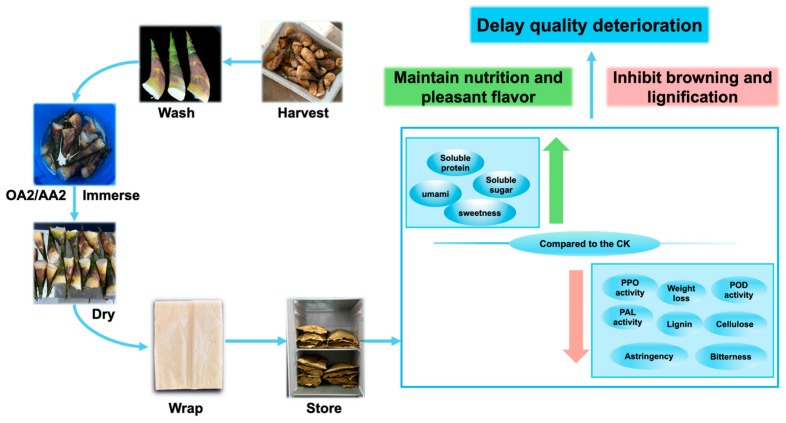
Postharvest OA2 and AA2 treatments delayed the quality deterioration of GBSs and LBSs and their regulation mechanisms. GBS, green bamboo shoot; LBS, lei bamboo shoot. OA2 represents samples treated with oxalic acid at 5 mmol·L^−1^; AA2 represents samples treated with ascorbic acid at 56.8 mmol·L^−1^. The green arrow indicates an increase in content compared to the CK; the red arrow indicates a decrease in content compared to the CK. The 12 blue gradient elliptical shapes respectively represent 8 physicochemical indicators and 4 flavor indicators.

Based on the quality index model, the AA2 treatment exhibited the optimal preservation effect for LBSs during the 14-day storage, which was consistent with the results of the PCA comprehensive evaluation model. However, for GBSs, a discrepancy was observed: the quality index model indicated the AA2 treatment as the most effective preservation method, whereas the PCA comprehensive evaluation model suggested that the OA2 treatment was superior. Therefore, a further comparative analysis was conducted to evaluate the preservation effects of the OA2 and AA2 treatments on GBSs. As illustrated in Figure 7, the comprehensive scores of OA2-treated GBS samples remained consistently lower than those of AA2-treated samples throughout the 14-day storage (difference > 0.1), indicating a pronounced difference between the two treatments, with OA2 showing better preservation according to PCA. Nevertheless, the Qi values of OA2- and AA2-treated GBS samples were nearly identical during days 3–10 of storage (difference < 0.001). From day 10 onward, the Qi values of AA2-treated samples began to slightly exceed those of OA2-treated samples (difference < 0.05), reaching a maximum difference of 0.04 by day 14. These results suggest that, based on the quality index model, the preservation effects of the OA2 and AA2 treatments on GBSs were not markedly different. As the PCA comprehensive evaluation model can more precisely compare the preservation effects of different treatments compared to the quality index model, it was employed in this study to select the best treatment for GBSs, leading to the conclusion that the OA2 treatment performed best during the 14-day storage. The regulation mechanisms of the OA2 and AA2 treatments delay the quality deterioration of GBSs and LBSs, as presented in Figure 8. Furthermore, these results indicate that the analytical approach for identifying the optimal preservation method for bamboo shoots may vary depending on the species. Therefore, it is necessary to develop a more universally applicable analytical method with enhanced accuracy for evaluating the comprehensive preservation quality of bamboo shoots in the future.

## 4. Conclusions

Postharvest organic acid treatments effectively preserved the preservation quality of both GBSs and LBSs by reducing weight loss; inhibiting the accumulation of lignin and cellulose; maintaining the higher levels of soluble protein, soluble sugar, sweetness and umami; and suppressing bitterness, astringency, and the activities of PAL, POD and PPO during 14 days of storage at 4 °C. By comparing the results obtained from the PCA comprehensive evaluation model and the quality index model, it was found that the quality index model recommended AA2 as the optimal treatment for both GBSs and LBSs, whereas the PCA comprehensive evaluation model identified variety-specific optimal treatment (OA2 for GBSs and AA2 for LBSs). Further discoveries indicated that the PCA comprehensive evaluation model provided a more accurate and discriminatory assessment of preservation efficacy for different bamboo shoot species. Comparative analysis further revealed that OA2-treated GBSs exhibited a longer shelf life than AA2-treated LBSs, highlighting the superior preservation effect of the OA2 treatment. In conclusion, this study demonstrated that 5 mmol·L^−1^ oxalic acid can serve as an effective and practical preservation strategy for bamboo shoots and that variety-specific selection of organic acid is essential. These findings provide valuable guidance for the bamboo shoot industry in tailoring preservation methods according to shoot species. Future studies should use methods such as transcriptomic and metabolomic analyses to explore the molecular mechanisms underlying the variety-specific responses and evaluate the commercial feasibility of the optimal treatments under industrial storage conditions. In addition, it is also necessary to conduct deeper studies, including microscopic observations, to further validate the structural changes in bamboo shoots during storage.

## Figures and Tables

**Table 1:** Effect of different organic acid treatments on soluble protein content of two bamboo shoot species.

**GBSs**				
**Treatment**	**Day 3 (mg/g)**	**Day 7 (mg/g)**	**Day 10 (mg/g)**	**Day 14 (mg/g)**
CK	17.72 ± 1.34 ^Aabcd^	14.94 ± 0.42 ^Bbcd^	13.92 ± 0.66 ^Bbcd^	11.36 ± 1.24 ^Ccd^
OA1	17.6 ± 0.62 ^Aabcd^	16.70 ± 1.03 ^Aa^	14.66 ± 1.15 ^Babc^	13.00 ± 0.64 ^Bab^
OA2	17.88 ± 0.81 ^Aabcd^	16.28 ± 0.92 ^Aabc^	14.97 ± 0.59 ^Bab^	11.98 ± 0.2 ^Cabcd^
OA3	18.60 ± 1.71 ^Aab^	16.11 ± 0.56 ^Babc^	13.14 ± 0.31 ^Ccde^	10.70 ± 0.9 ^Dd^
AA1	17.45 ± 1.33 ^Abcd^	14.23 ± 0.74 ^Bd^	13.28 ± 0.88 ^Bcde^	10.28 ± 0.78 ^Cd^
AA2	18.42 ± 0.95 ^Aabc^	15.32 ± 0.38 ^Bbcd^	14.38 ± 0.79 ^Babcd^	11.53 ± 0.65 ^Cbcd^
AA3	17.17 ± 1.26 ^Abcd^	15.79 ± 0.89 ^Babcd^	12.99 ± 0.81 ^Cde^	12.33 ± 0.34 ^Cabc^
CA1	16.66 ± 1.13 ^Ad^	14.83 ± 0.59 ^Bcd^	13.42 ± 1.01 ^Bcde^	11.37 ± 0.31 ^Ccd^
CA2	17.63 ± 0.88 ^Aabcd^	16.79 ± 0.36 ^Aa^	13.63 ± 0.75 ^Bbcde^	12.92 ± 0.89 ^Babc^
CA3	17.05 ± 1.08 ^Acd^	14.35 ± 0.69 ^Bd^	12.42 ± 0.02 ^Ce^	11.53 ± 1.16 ^Cbcd^
SA1	19.15 ± 1.31 ^Aa^	16.95 ± 1.28 ^Ba^	15.5 ± 0.68 ^Bab^	13.54 ± 0.87 ^Ca^
SA2	18.88 ± 1.41 ^Aab^	16.46 ± 0.56 ^Bab^	15.29 ± 1.02 ^Ba^	12.86 ± 0.57 ^Cabc^
SA3	17.97 ± 1.04 ^Aabcd^	16.65 ± 1.3 ^Ba^	15.29 ± 0.31 ^Ba^	12.81 ± 1.05 ^Cabc^
**LBSs**				
**Treatment**	**Day 3 (mg/g)**	**Day 7 (mg/g)**	**Day 10 (mg/g)**	**Day 14 (mg/g)**
CK	20.87 ± 0.85 ^Acdef^	18.09 ± 0.73 ^Bcde^	14.46 ± 0.26 ^Cbcd^	10.63 ± 0.34 ^Dcd^
OA1	22.9 ± 0.83 ^Aabc^	20.31 ± 0.87 ^Babc^	15.91 ± 0.56 ^Cabc^	11.73 ± 0.79 ^Dbcd^
OA2	23.73 ± 1.14 ^Aab^	21.44 ± 1.22 ^Ba^	15.5 ± 0.41 ^Cbc^	12.3 ± 0.94 ^Dabc^
OA3	19.71 ± 1.38 ^Aefg^	16.69 ± 0.89 ^Bde^	13.05 ± 0.74 ^Cde^	10.97 ± 0.80 ^Dbcd^
AA1	20.08 ± 0.58 ^Adef^	16.45 ± 0.41 ^Be^	15.4 ± 0.23 ^Cbcd^	10.35 ± 0.56 ^Dd^
AA2	23.95 ± 0.62 ^Aa^	21.75 ± 0.45 ^Ba^	17.12 ± 1.17 ^Ca^	13.59 ± 0.61 ^Dab^
AA3	22.18 ± 0.91 ^Abcde^	20.63 ± 0.83 ^Bab^	16.5 ± 0.81 ^Cab^	12.6 ± 0.14 ^Dab^
CA1	19.34 ± 0.72 ^Ag^	16.31 ± 0.53 ^Be^	13.32 ± 1.00 ^Cde^	10.32 ± 0.51 ^Dd^
CA2	23.9 ± 1.72 ^Aab^	18.99 ± 0.57 ^Bbcd^	15.88 ± 1.60 ^Cabc^	12.85 ± 1.11 ^Dab^
CA3	21.36 ± 1.37 ^Acdef^	18.22 ± 1.41 ^Bcde^	15.43 ± 0.70 ^Cbc^	13.94 ± 0.52 ^Ca^
SA1	19.61 ± 1.46 ^Afg^	16.85 ± 0.71 ^Bde^	13.77 ± 1.16 ^Cd^	12.1 ± 1.24 ^Dab^
SA2	19.34 ± 0.96 ^Afg^	17.61 ± 0.42 ^Bde^	14.6 ± 1.01 ^Cbcd^	12.59 ± 0.91 ^Dab^
SA3	22.31 ± 1.65 ^Abcd^	18.27 ± 0.37 ^Bcde^	15.15 ± 0.88 ^Cbc^	12.04 ± 1.05 ^Dabcd^

Note: GBSs, green bamboo shoots; LBSs, lei bamboo shoots. Results followed by different capital letters in the same row indicate differences during storage. Different lowercase letters in the same column indicate differences among the samples. CK represents control samples; OA1, OA2 and OA3 represent samples treated with oxalic acid at 2.5 mmol·L^−1^, 5 mmol·L^−1^ and 7.5 mmol·L^−1^, respectively; AA1, AA2 and AA3 represent samples treated with ascorbic acid at 28.4 mmol·L^−1^, 56.8 mmol·L^−1^, and 85.2 mmol·L^−1^, respectively; CA1, CA2 and CA3 represent samples treated with critic acid at 26 mmol·L^−1^, 52 mmol·L^−1^ and 78 mmol·L^−1^, respectively; SA1, SA2 and SA3 represent samples treated with salicylic acid at 0.5 mmol·L^−1^, 1 mmol·L^−1^ and 1.5 mmol·L^−1^, respectively.

**Table 2:** Effect of different organic acid treatments on soluble sugar contents of two bamboo shoot species.

**GBSs**				
**Treatment**	**Day 3 (%)**	**Day 7 (%)**	**Day 10 (%)**	**Day 14 (%)**
CK	1.46 ± 0.13 ^Ade^	1.1 ± 0.07 ^Bd^	0.93 ± 0.04 ^Cef^	0.6 ± 0.03 ^De^
OA1	1.72 ± 0.13 ^Aabc^	1.2 ± 0.07 ^Bd^	0.86 ± 0.03 ^Cf^	0.63 ± 0.03 ^Cde^
OA2	1.48 ± 0.03 ^Ade^	1.24 ± 0.07 ^Bcd^	1.05 ± 0.06 ^Cbcde^	0.64 ± 0.04 ^Dde^
OA3	1.54 ± 0.11 ^Acd^	1.21 ± 0.07 ^Bd^	1.03 ± 0.09 ^Ccde^	0.61 ± 0.02 ^Dde^
AA1	1.65 ± 0.14 ^Abcd^	1.2 ± 0.02 ^Bd^	1 ± 0.04 ^Cde^	0.82 ± 0.01 ^Dc^
AA2	1.78 ± 0.03 ^Aab^	1.4 ± 0.11 ^Bb^	1.14 ± 0.1 ^Cabc^	0.79 ± 0.03 ^Dc^
AA3	1.65 ± 0.05 ^Abcd^	1.38 ± 0.09 ^Bbc^	1.19 ± 0.05 ^Ca^	0.68 ± 0.05 ^Dd^
CA1	1.31 ± 0.11 ^Ae^	1.11 ± 0.12 ^Bd^	0.86 ± 0.08 ^Cf^	0.62 ± 0.04 ^Cde^
CA2	1.55 ± 0.12 ^Acd^	1.37 ± 0.06 ^Bbc^	0.94 ± 0.06 ^Cef^	0.6 ± 0.06 ^De^
CA3	1.47 ± 0.13 ^Ade^	1.19 ± 0.02 ^Bd^	1.09 ± 0.06 ^Babcd^	0.52 ± 0.02 ^Cf^
SA1	1.77 ± 0.15 ^Aab^	1.44 ± 0.09 ^Bb^	1.09 ± 0.02 ^Cabcd^	0.9 ± 0.02 ^Cb^
SA2	1.9 ± 0.11 ^Aa^	1.63 ± 0.06 ^Ba^	1.16 ± 0.04 ^Cab^	0.99 ± 0.03 ^Ca^
SA3	1.89 ± 0.08 ^Aa^	1.4 ± 0.05 ^Bb^	1.16 ± 0.1 ^Cab^	0.95 ± 0.06 ^Cab^
**LBSs**				
**Treatment**	**Day 3 (%)**	**Day 7 (%)**	**Day 10 (%)**	**Day 14 (%)**
CK	1.6 ± 0.04 ^Acdef^	1.31 ± 0.07 ^Befg^	1 ± 0.02 ^Ce^	0.83 ± 0.03 ^De^
OA1	1.67 ± 0.1 ^Abcd^	1.42 ± 0.07 ^Bcdef^	1.25 ± 0.08 ^Cabc^	1.07 ± 0.06 ^Dabc^
OA2	1.74 ± 0.09 ^Abc^	1.34 ± 0.11 ^Bef^	1.22 ± 0.02 ^Cabc^	1.06 ± 0.06 ^Dabc^
OA3	1.62 ± 0.03 ^Acde^	1.28 ± 0.03 ^Bfg^	1.18 ± 0.10 ^Bbcd^	0.97 ± 0.04 ^Cbcd^
AA1	1.45 ± 0.04 ^Aef^	1.3 ± 0.08 ^Befg^	1.09 ± 0.02 ^Ccde^	0.96 ± 0.06 ^Ccde^
AA2	1.41 ± 0.08 ^Af^	1.16 ± 0.03 ^Bg^	1.04 ± 0.03 ^Cde^	0.93 ± 0.08 ^Cde^
AA3	1.54 ± 0.03 ^Adef^	1.44 ± 0.03 ^Acde^	1.02 ± 0.06 ^Be^	0.96 ± 0.09 ^Bcde^
CA1	1.66 ± 0.09 ^Abcd^	1.42 ± 0.07 ^Bcdef^	1.16 ± 0.11 ^Cbcd^	1.06 ± 0.05 ^Cabc^
CA2	1.65 ± 0.06 ^Abcd^	1.5 ± 0.12 ^ABbcd^	1.33 ± 0.08 ^Bab^	1.09 ± 0.09 ^Cabc^
CA3	1.68 ± 0.16 ^Abcd^	1.4 ± 0.07 ^Bdef^	1.16 ± 0.11 ^Cbcd^	1.05 ± 0.06 ^Cabc^
SA1	1.83 ± 0.18 ^Aab^	1.64 ± 0.1 ^Bab^	1.33 ± 0.08 ^Cab^	1.11 ± 0.05 ^Da^
SA2	1.88 ± 0.05 ^Aa^	1.7 ± 0.08 ^Ba^	1.39 ± 0.08 ^Ca^	1.1 ± 0.10 ^Dab^
SA3	1.81 ± 0.15 ^Aab^	1.56 ± 0.07 ^Babc^	1.36 ± 0.03 ^Cab^	1.18 ± 0.04 ^Da^

Note: GBSs, green bamboo shoots; LBSs, lei bamboo shoots. Results followed by different capital letters in the same row indicate differences during storage. Different lowercase letters in the same column indicate differences among the samples. CK represents control samples; OA1, OA2 and OA3 represent samples treated with oxalic acid at 2.5 mmol·L^−1^, 5 mmol·L^−1^ and 7.5 mmol·L^−1^, respectively; AA1, AA2 and AA3 represent samples treated with ascorbic acid at 28.4 mmol·L^−1^, 56.8 mmol·L^−1^, and 85.2 mmol·L^−1^, respectively; CA1, CA2 and CA3 represent samples treated with critic acid at 26 mmol·L^−1^, 52 mmol·L^−1^ and 78 mmol·L^−1^, respectively; SA1, SA2 and SA3 represent samples treated with salicylic acid at 0.5 mmol·L^−1^, 1 mmol·L^−1^ and 1.5 mmol·L^−1^, respectively.

**Table 3:** Effect of different organic acid treatments on the weight loss of two bamboo shoot species.

**GBSs**				
**Treatment**	**Day 3 (%)**	**Day 7 (%)**	**Day 10 (%)**	**Day 14 (%)**
CK	4.01 ± 0.25 ^Dab^	11.27 ± 1.12 ^Cab^	17.12 ± 1.19 ^Bab^	27.9 ± 2.32 ^Aabc^
OA1	3.48 ± 0.25 ^Dbc^	8.37 ± 0.68 ^Cde^	12.73 ± 0.57 ^Bde^	22.82 ± 1.04 ^Aef^
OA2	3.67 ± 0.36 ^Dbc^	8.01 ± 0.25 ^Ce^	12.05 ± 0.43 ^Be^	21.74 ± 0.88 ^Af^
OA3	3.87 ± 0.3 ^Db^	9.5 ± 1.24 ^Cbc^	14.25 ± 0.28 ^Bcd^	23.84 ± 2.1 ^Adef^
AA1	3.45 ± 0.13 ^Db^	9.07 ± 0.67 ^Ccd^	15.88 ± 1.06 ^Bbc^	24.69 ± 0.83 ^Acde^
AA2	3.81 ± 0.22 ^Db^	8.23 ± 0.37 ^Cde^	14.85 ± 0.78 ^Bc^	24.46 ± 1.05 ^Ade^
AA3	3.87 ± 0.30 ^Db^	11.79 ± 1.05 ^Ca^	18.86 ± 0.62 ^Ba^	29.94 ± 0.7 ^Aa^
CA1	3.91 ± 0.30 ^Db^	10.02 ± 0.70 ^Cb^	14.7 ± 0.78 ^Bc^	25.13 ± 1.52 ^Abcde^
CA2	4.04 ± 0.17 ^Dab^	9.12 ± 0.40 ^Ccd^	13.11 ± 0.25 ^Bcde^	24.89 ± 1.36 ^Abcde^
CA3	3.15 ± 0.25 ^Dc^	9.10 ± 0.45 ^Ccd^	13.47 ± 0.89 ^Bcde^	24.84 ± 0.95 ^Abcde^
SA1	3.21 ± 0.39 ^Dc^	9.43 ± 0.79 ^Cbc^	16.06 ± 0.48 ^Babc^	26.41 ± 1.64 ^Abcd^
SA2	4.01 ± 0.30 ^Dab^	9.07 ± 0.59 ^Ccd^	15.68 ± 0.92 ^Bbc^	24.63 ± 1.10 ^Acde^
SA3	4.3 ± 0.28 ^Da^	10.50 ± 0.23 ^Cab^	18.22 ± 0.42 ^Ba^	28.21 ± 2.21 ^Aab^
**LBSs**				
**Treatment**	**Day 3 (%)**	**Day 7 (%)**	**Day 10 (%)**	**Day 14 (%)**
CK	4.11 ± 0.32 ^Dab^	10.66 ± 0.37 ^Cbcd^	15.11 ± 0.29 ^Bbc^	18.12 ± 0.15 ^Abc^
OA1	4.07 ± 0.66 ^Cab^	12.89 ± 0.25 ^Bab^	14.34 ± 0.23 ^Bbcd^	17.73 ± 0.46 ^Acd^
OA2	4.38 ± 0.30 ^Dab^	10.67 ± 0.46 ^Cbcd^	13.10 ± 0.25 ^Bcde^	16.65 ± 0.11 ^Acde^
OA3	3.95 ± 0.17 ^Dab^	8.84 ± 0.18 ^Cdef^	12.20 ± 0.56 ^Bcde^	15.62 ± 0.20 ^Adef^
AA1	3.53 ± 0.01 ^Db^	7.95 ± 0.37 ^Cef^	11.61 ± 0.24 ^Bcde^	15.24 ± 0.32 ^Aef^
AA2	3.62 ± 0.18 ^Db^	7.26 ± 0.22 ^Cf^	10.74 ± 0.62 ^Be^	14.22 ± 0.23 ^Af^
AA3	3.66 ± 0.29 ^Dab^	9.31 ± 0.13 ^Cdef^	13.07 ± 0.06 ^Bcde^	17.43 ± 0.25 ^Acde^
CA1	4.73 ± 0.23 ^Dab^	10.24 ± 0.08 ^Bcde^	11.78 ± 0.21 ^Bcde^	15.54 ± 0.26 ^Adef^
CA2	4.16 ± 0.15 ^Dab^	7.94 ± 0.21 ^Cef^	10.83 ± 0.30 ^Bde^	16.67 ± 0.30 ^Acde^
CA3	3.79 ± 0.15 ^Dab^	8.62 ± 0.45 ^Cdef^	13.80 ± 0.82 ^Bbcde^	16.89 ± 0.26 ^Acde^
SA1	4.74 ± 0.69 ^Dab^	12.39 ± 0.20 ^Cabc^	17.21 ± 0.36 ^Bab^	21.46 ± 0.23 ^Aa^
SA2	5.35 ± 0.12 ^Da^	14.58 ± 0.25 ^Ca^	20.41 ± 0.73 ^Ba^	22.10 ± 0.44 ^Aa^
SA3	5.17 ± 0.27 ^Dab^	13.34 ± 0.35 ^Ca^	19.02 ± 0.20 ^Aa^	20.41 ± 0.33 ^Aab^

Note: GBSs, green bamboo shoots; LBSs, lei bamboo shoots. Results followed by different capital letters in the same row indicate differences during storage. Different lowercase letters in the same column indicate differences among the samples. CK represents control samples; OA1, OA2 and OA3 represent samples treated with oxalic acid at 2.5 mmol·L^−1^, 5 mmol·L^−1^ and 7.5 mmol·L^−1^, respectively; AA1, AA2 and AA3 represent samples treated with ascorbic acid at 28.4 mmol·L^−1^, 56.8 mmol·L^−1^, and 85.2 mmol·L^−1^, respectively; CA1, CA2 and CA3 represent samples treated with critic acid at 26 mmol·L^−1^, 52 mmol·L^−1^ and 78 mmol·L^−1^, respectively; SA1, SA2 and SA3 represent samples treated with salicylic acid at 0.5 mmol·L^−1^, 1 mmol·L^−1^ and 1.5 mmol·L^−1^, respectively.

**Table 4:** Effect of different organic acid treatments on the lignin content of two bamboo shoot species.

**GBSs**				
**Treatment**	**Day 3 (%)**	**Day 7 (%)**	**Day 10 (%)**	**Day 14 (%)**
CK	0.55 ± 0.04 ^Da^	0.9 ± 0.04 ^Ca^	1.21 ± 0.05 ^Ba^	1.44 ± 0.11 ^Aa^
OA1	0.39 ± 0.03 ^Ccde^	0.49 ± 0.04 ^Cg^	0.91 ± 0.06 ^Bc^	1.16 ± 0.15 ^Ac^
OA2	0.38 ± 0.03 ^Bde^	0.57 ± 0.04 ^Bdef^	0.95 ± 0.05 ^Abc^	1.18 ± 0.09 ^Aabc^
OA3	0.33 ± 0.03 ^De^	0.62 ± 0.05 ^Ccde^	1.01 ± 0.01 ^Bbc^	1.24 ± 0.09 ^Abc^
AA1	0.35 ± 0.02 ^Be^	0.55 ± 0.04 ^Befg^	1.01 ± 0.1 ^Abc^	1.17 ± 0.04 ^Ac^
AA2	0.33 ± 0.03 ^Ce^	0.51 ± 0.03 ^Cfg^	1.04 ± 0.09 ^Bbc^	1.35 ± 0.14 ^Aab^
AA3	0.5 ± 0.06 ^Cab^	0.84 ± 0.02 ^Ba^	1.02 ± 0.03 ^Bbc^	1.19 ± 0.06 ^Abc^
CA1	0.35 ± 0.01 ^De^	0.63 ± 0.06 ^Ccd^	1.01 ± 0.05 ^Bbc^	1.28 ± 0.08 ^Aabc^
CA2	0.44 ± 0.05 ^Cbcd^	0.6 ± 0.03 ^Cde^	0.99 ± 0.09 ^Bbc^	1.23 ± 0.02 ^Abc^
CA3	0.47 ± 0.05 ^Bb^	0.63 ± 0.03 ^Bcde^	1.09 ± 0.1 ^Aab^	1.2 ± 0.11 ^Abc^
SA1	0.46 ± 0.04 ^Dbc^	0.72 ± 0.02 ^Cb^	1.03 ± 0.06 ^Bbc^	1.24 ± 0.06 ^Abc^
SA2	0.38 ± 0.01 ^Dde^	0.68 ± 0.03 ^Cbc^	1.05 ± 0.06 ^Bb^	1.35 ± 0.06 ^Aab^
SA3	0.46 ± 0.05 ^Dbc^	0.74 ± 0.04 ^Cb^	1.02 ± 0.09 ^Bbc^	1.29 ± 0.15 ^Aabc^
**LBSs**				
**Treatment**	**Day 3 (%)**	**Day 7 (%)**	**Day 10 (%)**	**Day 14 (%)**
CK	0.44 ± 0.04 ^Da^	0.78 ± 0.06 ^Ca^	1.12 ± 0.03 ^Ba^	1.43 ± 0.14 ^Aa^
OA1	0.33 ± 0.03 ^Cdef^	0.6 ± 0.05 ^Bbc^	0.65 ± 0.03 ^Bef^	0.93 ± 0.04 ^Adef^
OA2	0.27 ± 0.03 ^Cf^	0.54 ± 0.03 ^Bcd^	0.77 ± 0.07 ^Acde^	0.85 ± 0.07 ^Af^
OA3	0.29 ± 0.01 ^Cef^	0.66 ± 0.05 ^Bb^	0.79 ± 0.08 ^Bbcd^	0.94 ± 0.08 ^Adef^
AA1	0.33 ± 0.03 ^Cdef^	0.66 ± 0.03 ^Bb^	0.77 ± 0.04 ^ABcde^	0.9 ± 0.07 ^Aef^
AA2	0.29 ± 0.02 ^Cef^	0.5 ± 0.06 ^Bd^	0.62 ± 0.06 ^ABf^	0.8 ± 0.04 ^Af^
AA3	0.41 ± 0.03 ^Da^	0.67 ± 0.05 ^Cb^	1.08 ± 0.09 ^Ba^	1.33 ± 0.16 ^Aab^
CA1	0.33 ± 0.04 ^Ddef^	0.67 ± 0.02 ^Cb^	0.86 ± 0.05 ^Bbc^	1.09 ± 0.08 ^Acd^
CA2	0.35 ± 0.04 ^Dbcd^	0.55 ± 0.04 ^Ccd^	0.71 ± 0.06 ^Bdef^	1.03 ± 0.04 ^Ade^
CA3	0.35 ± 0.04 ^Dbcd^	0.6 ± 0.04 ^Cbc^	0.9 ± 0.07 ^Bb^	1.21 ± 0.08 ^Abc^
SA1	0.32 ± 0.02 ^Ddef^	0.59 ± 0.04 ^Cbc^	0.84 ± 0.09 ^Bbc^	1.05 ± 0.08 ^Acde^
SA2	0.39 ± 0.01 ^Dabc^	0.67 ± 0.08 ^Cb^	0.87 ± 0.08 ^Bbc^	1.08 ± 0.04 ^Acd^
SA3	0.41 ± 0.03 ^Dab^	0.66 ± 0.03 ^Cb^	0.9 ± 0.03 ^Bb^	1.22 ± 0.09 ^Abc^

Note: GBSs, green bamboo shoots; LBSs, lei bamboo shoots. Results followed by different capital letters in the same row indicate differences during storage. Different lowercase letters in the same column indicate differences among the samples. CK represents control samples; OA1, OA2 and OA3 represent samples treated with oxalic acid at 2.5 mmol·L^−1^, 5 mmol·L^−1^ and 7.5 mmol·L^−1^, respectively; AA1, AA2 and AA3 represent samples treated with ascorbic acid at 28.4 mmol·L^−1^, 56.8 mmol·L^−1^, and 85.2 mmol·L^−1^, respectively; CA1, CA2 and CA3 represent samples treated with critic acid at 26 mmol·L^−1^, 52 mmol·L^−1^ and 78 mmol·L^−1^, respectively; SA1, SA2 and SA3 represent samples treated with salicylic acid at 0.5 mmol·L^−1^, 1 mmol·L^−1^ and 1.5 mmol·L^−1^, respectively.

**Table 5:** Effect of different organic acid treatments on the cellulose content of two bamboo shoot species.

**GBSs**				
**Treatment**	**Day3 (%)**	**Day7 (%)**	**Day10 (%)**	**Day14 (%)**
CK	28.32 ± 0.50 ^Da^	43.79 ± 1.22 ^Ca^	52.72 ± 1.36 ^Ba^	58.81 ± 2.04 ^Aa^
OA1	18.60 ± 0.80 ^Cf^	30.48 ± 0.58 ^Bf^	41.13 ± 0.88 ^Acd^	45.79 ± 0.48 ^Af^
OA2	19.76 ± 1.62 ^Def^	31.15 ± 1.82 ^Cdef^	40.56 ± 1.37 ^Bd^	49.39 ± 1.58 ^Adef^
OA3	20.09 ± 1.38 ^Ddef^	32.71 ± 0.72 ^Ccde^	41.95 ± 1.18 ^Bcd^	48.32 ± 2.48 ^Aef^
AA1	19.84 ± 0.62 ^Def^	32.09 ± 0.67 ^Cdef^	44.51 ± 0.69 ^Bbcd^	55.15 ± 3.42 ^Aab^
AA2	18.99 ± 0.75 ^Df^	35.43 ± 0.67 ^Ccd^	46.77 ± 1.43 ^Bb^	52.53 ± 3.1 ^Abcde^
AA3	22.28 ± 1.28 ^Dbcd^	34.06 ± 1.39 ^Ccd^	47.99 ± 1.61 ^Bb^	54.63 ± 2.28 ^Aabc^
CA1	20.47 ± 1.25 ^Ddef^	33.13 ± 1.24 ^Cdef^	44.57 ± 2.20 ^Bbc^	52.52 ± 0.94 ^Abcde^
CA2	19.76 ± 1.36 ^Def^	31.69 ± 0.4 ^Cef^	43.57 ± 1.72 ^Bcd^	53.92 ± 4.39 ^Aabcd^
CA3	23.61 ± 1.34 ^Dbc^	31.27 ± 1.17 ^Cf^	44.75 ± 2.09 ^Bbc^	53.42 ± 1.01 ^Abcd^
SA1	23.57 ± 1.64 ^Cbc^	38.62 ± 0.77 ^Bb^	49.00 ± 1.00 ^Ab^	53.9 ± 2.06 ^Aabcd^
SA2	21.40 ± 0.71 ^Bcde^	36.61 ± 0.78 ^Bbc^	47.88 ± 1.18 ^Ab^	49.83 ± 0.84 ^Acdef^
SA3	24.10 ± 0.97 ^Db^	37.72 ± 1.58 ^Cbc^	45.73 ± 1.50 ^Bbc^	53.99 ± 3.77 ^Aabcd^
**LBSs**				
**Treatment**	**Day 3 (%)**	**Day 7 (%)**	**Day 10 (%)**	**Day 14 (%)**
CK	21.66 ± 1.38 ^Da^	34.5 ± 2.6 ^Ca^	43.2 ± 1.78 ^Ba^	49.7 ± 1.41 ^Aa^
OA1	14.26 ± 0.97 ^De^	28.65 ± 2.53 ^Cbcde^	36.52 ± 1.69 ^Bde^	42.86 ± 1.03 ^Acde^
OA2	15.34 ± 0.83 ^Dcde^	27.14 ± 1.57 ^Cdef^	38.01 ± 1.23 ^Bde^	43.56 ± 1.68 ^Acde^
OA3	17.51 ± 0.88 ^Cbcde^	32.57 ± 1.88 ^Bab^	41.62 ± 0.87 ^Aabc^	44.26 ± 1.25 ^Abcd^
AA1	15.10 ± 1.12 ^Dcde^	24.85 ± 1.10 ^Cef^	34.49 ± 1.25 ^Be^	40.06 ± 1.11 ^Aef^
AA2	14.37 ± 1.09 ^Ce^	23.73 ± 0.74 ^Bf^	34.82 ± 3.42 ^Ae^	38.15 ± 0.15 ^Af^
AA3	16.14 ± 1.06 ^Ccde^	27.35 ± 2.42 ^Bdef^	36.52 ± 1.15 ^Ade^	39.26 ± 0.56 ^Af^
CA1	15.48 ± 1.38 ^Ccde^	32.09 ± 1.02 ^BCab^	36.77 ± 0.48 ^Bde^	47.4 ± 1.38 ^Aab^
CA2	14.18 ± 1.44 ^De^	31.51 ± 2.28 ^Cabc^	38.56 ± 1.72 ^Bcd^	48.64 ± 0.55 ^Aa^
CA3	18.86 ± 1.19 ^Cbcd^	27.71 ± 0.67 ^Bcdef^	38.57 ± 1.08 ^Acd^	42.22 ± 1.14 ^Ade^
SA1	19.24 ± 1.58 ^Dbc^	32.19 ± 2.76 ^Cab^	42.21 ± 1.72 ^Bab^	47.28 ± 2.79 ^Aab^
SA2	15.43 ± 0.87 ^Dcde^	29.99 ± 1.78 ^Cbcd^	39.08 ± 2.03 ^Bbcd^	43.6 ± 1.51 ^Acde^
SA3	20.90 ± 1.20 ^Dab^	30.49 ± 2.67 ^Cabcd^	34.72 ± 2.29 ^Be^	44.22 ± 0.24 ^Abcd^

Note: GBSs, green bamboo shoots; LBSs, lei bamboo shoots. Results followed by different capital letters in the same row indicate differences during storage. Different lowercase letters in the same column indicate differences among the samples. CK represents control samples; OA1, OA2 and OA3 represent samples treated with oxalic acid at 2.5 mmol·L^−1^, 5 mmol·L^−1^ and 7.5 mmol·L^−1^, respectively; AA1, AA2 and AA3 represent samples treated with ascorbic acid at 28.4 mmol·L^−1^, 56.8 mmol·L^−1^, and 85.2 mmol·L^−1^, respectively; CA1, CA2 and CA3 represent samples treated with critic acid at 26 mmol·L^−1^, 52 mmol·L^−1^ and 78 mmol·L^−1^, respectively; SA1, SA2 and SA3 represent samples treated with salicylic acid at 0.5 mmol·L^−1^, 1 mmol·L^−1^ and 1.5 mmol·L^−1^, respectively.

**Table 6:** Effect of different organic acid treatments on PAL activity of two bamboo shoot species.

**GBSs**				
**Treatment**	**Day 3 (U/g∙min)**	**Day 7 (U/g∙min)**	**Day 10 (U/g∙min)**	**Day 14 (U/g∙min)**
CK	44.80 ± 2.89 ^Da^	58.94 ± 1.46 ^Ca^	70.52 ± 3.79 ^Bab^	81.40 ± 1.65 ^Aa^
OA1	31.33 ± 1.26 ^Dde^	43.87 ± 3.51 ^Cde^	55.36 ± 2.51 ^Bef^	65.63 ± 0.54 ^Abc^
OA2	29.65 ± 1.18 ^De^	36.04 ± 2.44 ^Cf^	50.47 ± 2.72 ^Bf^	62.56 ± 3.30 ^Ac^
OA3	32.03 ± 1.39 ^Dd^	38.29 ± 2.86 ^Cef^	49.37 ± 3.74 ^Bf^	69.15 ± 1.36 ^Aabc^
AA1	34.94 ± 1.13 ^Dcd^	46.38 ± 3.09 ^Ccd^	63.20 ± 1.67 ^Bbcd^	70.70 ± 2.57 ^Aabc^
AA2	35.23 ± 0.94 ^Dcd^	47.80 ± 2.28 ^Cbcd^	58.16 ± 0.93 ^Bde^	69.34 ± 3.32 ^Aabc^
AA3	34.66 ± 2.00 ^Cd^	49.04 ± 0.24 ^Bbcd^	64.78 ± 3.07 ^Abcd^	69.60 ± 3.33 ^Aabc^
CA1	38.81 ± 1.93 ^Cbc^	52.67 ± 3.76 ^Babc^	73.51 ± 3.25 ^Aa^	75.93 ± 1.98 ^Aab^
CA2	41.14 ± 1.24 ^Dab^	54.14 ± 3.73 ^Cab^	67.40 ± 3.53 ^Babc^	73.23 ± 1.24 ^Aabc^
CA3	41.32 ± 2.98 ^Dab^	51.55 ± 3.20 ^Cbc^	60.62 ± 3.09 ^Bcde^	71.86 ± 2.62 ^Aabc^
SA1	33.71 ± 2.54 ^Dd^	42.44 ± 2.26 ^Cdef^	59.16 ± 3.54 ^Bde^	69.22 ± 1.91 ^Aabc^
SA2	33.90 ± 1.79 ^Dd^	42.64 ± 3.54 ^Cdef^	55.51 ± 2.42 ^Bef^	68.80 ± 2.82 ^Aabc^
SA3	34.11 ± 2.29 ^Dd^	47.12 ± 3.38 ^Ccd^	60.11 ± 3.29 ^Bcde^	73.06 ± 2.57 ^Aabc^
**LBSs**				
**Treatment**	**Day 3**	**Day 7**	**Day 10**	**Day 14**
CK	49.29 ± 1.61 ^Da^	64.48 ± 1.56 ^Cab^	89.03 ± 6.24 ^Aa^	71.89 ± 1.65 ^Bcd^
OA1	43.11 ± 1.62 ^Dcde^	57.82 ± 4.51 ^Cc^	77.68 ± 5.21 ^Bbc^	86.03 ± 4.64 ^Aa^
OA2	45.31 ± 3.42 ^Dabc^	60.01 ± 1.49 ^Cbc^	74.95 ± 1.11 ^Bbcd^	79.77 ± 5.78 ^Aabc^
OA3	44.13 ± 1.57 ^Dbcd^	59.81 ± 0.77 ^Cbc^	83.12 ± 4.39 ^Aabc^	75.77 ± 0.85 ^Bbcd^
AA1	42.87 ± 1.03 ^Dcde^	54.76 ± 5.3 ^Ccd^	76.11 ± 3.16 ^Bbc^	81.87 ± 3.24 ^Aab^
AA2	37.63 ± 2.35 ^Df^	49.73 ± 2.61 ^Cd^	67.37 ± 6.18 ^Be^	76.94 ± 3.31 ^Abcd^
AA3	38.86 ± 1.76 ^Def^	55.7 ± 4.2 ^Ccd^	69.55 ± 3.33 ^Bde^	80.59 ± 3.84 ^Aab^
CA1	44.37 ± 1.2 ^Dbcd^	67.84 ± 0.68 ^Ca^	84.11 ± 3.73 ^Aabc^	73.75 ± 4.3 ^Bcd^
CA2	46.02 ± 3.8 ^Dabc^	64.36 ± 2.38 ^Cab^	81.1 ± 2.94 ^Aabc^	70.4 ± 3.18 ^Bd^
CA3	47.89 ± 3.06 ^Dab^	67.98 ± 4.82 ^Ba^	86.18 ± 4.89 ^Aab^	71.23 ± 2.38 ^Bd^
SA1	45.26 ± 0.43 ^Dabc^	66.2 ± 2.6 ^Bab^	70.6 ± 4.31 ^Bde^	77.67 ± 5.29 ^Abcd^
SA2	40.63 ± 2.9 ^Ddef^	60.03 ± 3.5 ^Cbc^	67.96 ± 0.92 ^Be^	79.37 ± 4.07 ^Aabc^
SA3	42.03 ± 2.17 ^Dcdef^	56.99 ± 2.24 ^Cc^	70.94 ± 4.35 ^Bde^	81.64 ± 2.82 ^Aab^

Note: GBSs, green bamboo shoots; LBSs, lei bamboo shoots. Results followed by different capital letters in the same row indicate differences during storage. Different lowercase letters in the same column indicate differences among the samples. CK represents control samples; OA1, OA2 and OA3 represent samples treated with oxalic acid at 2.5 mmol·L^−1^, 5 mmol·L^−1^ and 7.5 mmol·L^−1^, respectively; AA1, AA2 and AA3 represent samples treated with ascorbic acid at 28.4 mmol·L^−1^, 56.8 mmol·L^−1^, and 85.2 mmol·L^−1^, respectively; CA1, CA2 and CA3 represent samples treated with critic acid at 26 mmol·L^−1^, 52 mmol·L^−1^ and 78 mmol·L^−1^, respectively; SA1, SA2 and SA3 represent samples treated with salicylic acid at 0.5 mmol·L^−1^, 1 mmol·L^−1^ and 1.5 mmol·L^−1^, respectively.

**Table 7:** Effect of different organic acid treatments on POD activity of two bamboo shoot species.

**GBSs**				
**Treatment**	**Day 3 (U/g∙min)**	**Day 7 (U/g∙min)**	**Day 10 (U/g∙min)**	**Day 14 (U/g∙min)**
CK	26.56 ± 0.86 ^Da^	40.66 ± 3.66 ^Ca^	54.47 ± 2.03 ^Ba^	72.35 ± 0.48 ^Aa^
OA1	19.43 ± 1.14 ^Cefg^	26.75 ± 2.44 ^Cfg^	40.04 ± 2.02 ^Bcd^	58.49 ± 1.67 ^Adef^
OA2	18.59 ± 0.39 ^Cfg^	24.33 ± 2.06 ^Cg^	39.1 ± 1.4 ^Bcd^	51.19 ± 1.53 ^Ag^
OA3	20.65 ± 0.59 ^Ddef^	29.92 ± 1.88 ^Cef^	42.83 ± 1.55 ^Bbc^	56.03 ± 3.1 ^Aefg^
AA1	22.26 ± 1.39 ^Cbcd^	34.15 ± 1.48 ^Bcd^	39.83 ± 0.55 ^Bcd^	59.81 ± 2.1 ^Acdef^
AA2	18.48 ± 0.4 ^Dg^	28.65 ± 0.71 ^Cef^	39.65 ± 3.28 ^Bcd^	54.46 ± 2.16 ^Afg^
AA3	24.04 ± 0.75 ^Db^	38.28 ± 2.02 ^Cab^	45.5 ± 2.39 ^Bb^	65.47 ± 3.34 ^Ab^
CA1	21.45 ± 1.17 ^Dcde^	31.72 ± 1.94 ^Cde^	45.8 ± 0.96 ^Bb^	63.29 ± 4.89 ^Abcd^
CA2	20.3 ± 1.19 ^Ddefg^	32.43 ± 1.9 ^Ccde^	41.79 ± 1.63 ^Bbcd^	55.87 ± 2.68 ^Aefg^
CA3	18.98 ± 1.36 ^Cfg^	36.07 ± 0.93 ^Bbc^	42.58 ± 3.22 ^Bbc^	57.39 ± 3.25 ^Aef^
SA1	18.89 ± 0.84 ^Dfg^	30.21 ± 2.28 ^Cdef^	41.58 ± 2.71 ^Bbcd^	60.62 ± 1.54 ^Abcde^
SA2	18.82 ± 0.98 ^Dfg^	32.52 ± 1.4 ^Ccde^	42.17 ± 2.69 ^Bbc^	56.26 ± 2.26 ^Aefg^
SA3	22.46 ± 1.43 ^Cbc^	26.41 ± 1.15 ^Cfg^	44.41 ± 0.88 ^Bb^	65.39 ± 3.89 ^Abc^
**LBSs**				
**Treatment**	**Day 3**	**Day 7**	**Day 10**	**Day 14**
CK	26.24 ± 0.98 ^Dab^	48.72 ± 2.20 ^Ca^	63.88 ± 1.17 ^Ba^	80.43 ± 4.61 ^Aa^
OA1	23.42 ± 1.35 ^Dc^	36.85 ± 2.93 ^Cbcd^	55.88 ± 2.18 ^Bbcd^	74.88 ± 4.00 ^Abc^
OA2	23.47 ± 0.76 ^Dc^	35.34 ± 1.64 ^Ccde^	57.38 ± 4.99 ^Babc^	65.1 ± 5.66 ^Adef^
OA3	26.52 ± 0.87 ^Da^	34.91 ± 2.21 ^Ccdef^	49.15 ± 3.53 ^Bde^	56.02 ± 1.32 ^Af^
AA1	20.49 ± 0.97 ^Dde^	33.35 ± 1.05 ^Cdef^	50.50 ± 3.98 ^Bcde^	65.46 ± 4.87 ^Acdef^
AA2	19.10 ± 0.77 ^De^	30.97 ± 1.45 ^Cf^	49.48 ± 3.35 ^Bde^	60.91 ± 5.16 ^Aef^
AA3	19.21 ± 0.92 ^De^	32.58 ± 2.09 ^Cef^	46.55 ± 3.01 ^Be^	58.63 ± 3.42 ^Af^
CA1	22.76 ± 0.92 ^Dc^	33.80 ± 3.06 ^Cdef^	55.16 ± 3.81 ^Bbcd^	71.44 ± 3.60 ^Abcd^
CA2	23.29 ± 0.69 ^Dc^	34.78 ± 0.65 ^Ccdef^	59.65 ± 2.10 ^Bab^	71.19 ± 3.18 ^Abcd^
CA3	23.39 ± 1.12 ^Dc^	38.71 ± 1.51 ^Cbc^	61.48 ± 5.42 ^Bab^	68.88 ± 3.75 ^Acde^
SA1	23.50 ± 1.17 ^Dbc^	41.90 ± 2.85 ^Cb^	60.00 ± 4.48 ^Bab^	71.11 ± 2.79 ^Abc^
SA2	21.85 ± 0.17 ^Dcd^	36.23 ± 1.32 ^Cbcd^	49.71 ± 3.0 ^Bde^	66.06 ± 4.21 ^Acde^
SA3	22.75 ± 0.42 ^Dc^	39.16 ± 2.55 ^Cb^	59.18 ± 2.53 ^Bab^	71.53 ± 2.41 ^Abcd^

Note: GBSs, green bamboo shoots; LBSs, lei bamboo shoots. Results followed by different capital letters in the same row indicate differences during storage. Different lowercase letters in the same column indicate differences among the samples. CK represents control samples; OA1, OA2 and OA3 represent samples treated with oxalic acid at 2.5 mmol·L^−1^, 5 mmol·L^−1^ and 7.5 mmol·L^−1^, respectively; AA1, AA2 and AA3 represent samples treated with ascorbic acid at 28.4 mmol·L^−1^, 56.8 mmol·L^−1^, and 85.2 mmol·L^−1^, respectively; CA1, CA2 and CA3 represent samples treated with critic acid at 26 mmol·L^−1^, 52 mmol·L^−1^ and 78 mmol·L^−1^, respectively; SA1, SA2 and SA3 represent samples treated with salicylic acid at 0.5 mmol·L^−1^, 1 mmol·L^−1^ and 1.5 mmol·L^−1^, respectively.

**Table 8:** Effect of different organic acid treatments on PPO activity of two bamboo shoot species.

**GBSs**				
**Treatment**	**Day 3 (U/g∙min)**	**Day 7 (U/g∙min)**	**Day 10 (U/g∙min)**	**Day 14 (U/g∙min)**
CK	1.48 ± 0.1 ^Ca^	1.84 ± 0.04 ^Ba^	2.02 ± 0.07 ^Aa^	1.98 ± 0.09 ^Aab^
OA1	1.22 ± 0.02 ^Cbc^	1.39 ± 0.04 ^Bdef^	1.76 ± 0.03 ^Abcd^	1.89 ± 0.09 ^Aabc^
OA2	1.16 ± 0.07 ^Dc^	1.38 ± 0.03 ^Cef^	1.58 ± 0.07 ^Bd^	1.75 ± 0.02 ^Acd^
OA3	1.22 ± 0.05 ^Cbc^	1.36 ± 0.01 ^Bf^	1.64 ± 0.08 ^Acd^	1.7 ± 0.06 ^Ad^
AA1	1.31 ± 0.07 ^Db^	1.66 ± 0.02 ^Cb^	1.83 ± 0.08 ^Babc^	2.05 ± 0.04 ^Aa^
AA2	1.24 ± 0.09 ^Cbc^	1.52 ± 0.07 ^Bcde^	1.75 ± 0.04 ^Abcd^	1.75 ± 0.03 ^Acd^
AA3	1.26 ± 0.1 ^Dbc^	1.62 ± 0.02 ^Cbc^	1.99 ± 0.02 ^Aa^	1.85 ± 0.1 ^Bbcd^
CA1	1.27 ± 0.03 ^Db^	1.54 ± 0.1 ^Cbcd^	1.73 ± 0.02 ^Bbcd^	1.92 ± 0.06 ^Aabc^
CA2	1.3 ± 0.05 ^Bb^	1.42 ± 0.02 ^Bdef^	1.69 ± 0.02 ^Acd^	1.81 ± 0.07 ^Abcd^
CA3	1.23 ± 0.08 ^Db^	1.4 ± 0.06 ^Cdef^	1.71 ± 0.05 ^Bbcd^	1.9 ± 0.05 ^Aabc^
SA1	1.22 ± 0.07 ^Cbc^	1.56 ± 0.02 ^Bbcd^	1.92 ± 0.04 ^Aab^	1.94 ± 0.1 ^Aab^
SA2	1.24 ± 0.08 ^Cbc^	1.43 ± 0.09 ^Bdef^	1.76 ± 0.03 ^Abcd^	1.75 ± 0.04 ^Acd^
SA3	1.22 ± 0.06 ^Dbc^	1.46 ± 0.06 ^Ccde^	1.74 ± 0.01 ^Bbcd^	1.98 ± 0.07 ^Aab^
**LBSs**				
**Treatment**	**Day 3**	**Day 7**	**Day 10**	**Day 14**
CK	1.68 ± 0.06 ^Ca^	2.03 ± 0.03 ^Aa^	2.15 ± 0.11 ^Aa^	1.85 ± 0.11 ^Bab^
OA1	1.53 ± 0.06 ^Cab^	1.76 ± 0.06 ^Bb^	1.91 ± 0.1 ^ABab^	2.05 ± 0.03 ^Aa^
OA2	1.39 ± 0.03 ^Cc^	1.59 ± 0.03 ^Bc^	1.71 ± 0.11 ^ABc^	1.89 ± 0.06 ^Aab^
OA3	1.61 ± 0.04 ^Cab^	1.85 ± 0.12 ^Bab^	2.07 ± 0.03 ^Aa^	1.9 ± 0.01 ^ABab^
AA1	1.46 ± 0.05 ^Cbc^	1.66 ± 0.09 ^Bbc^	1.82 ± 0.16 ^ABbc^	1.94 ± 0.12 ^Aab^
AA2	1.42 ± 0.04 ^Cbc^	1.57 ± 0.02 ^Bc^	1.73 ± 0.1 ^ABc^	1.81 ± 0.06 ^Ab^
AA3	1.48 ± 0.03 ^Cbc^	1.77 ± 0.08 ^ABb^	1.96 ± 0.12 ^Aab^	1.89 ± 0.09 ^Aab^
CA1	1.59 ± 0.09 ^Cab^	1.83 ± 0.1 ^ABab^	2.01 ± 0.08 ^Aab^	1.96 ± 0.1 ^Aab^
CA2	1.54 ± 0.07 ^Cab^	1.71 ± 0.01 ^Bbc^	1.87 ± 0.12 ^ABbc^	2.06 ± 0.12 ^Aa^
CA3	1.49 ± 0.11 ^Cbc^	1.63 ± 0.08 ^Bbc^	1.85 ± 0.11 ^ABbc^	1.98 ± 0.12 ^Aab^
SA1	1.51 ± 0.08 ^Cab^	1.79 ± 0.08 ^Bb^	1.91 ± 0.07 ^ABab^	2.02 ± 0.08 ^Aa^
SA2	1.43 ± 0.06 ^Cbc^	1.7 ± 0.07 ^Bbc^	1.83 ± 0.04 ^ABbc^	1.97 ± 0.08 ^Aab^
SA3	1.42 ± 0.07 ^Cbc^	1.67 ± 0.08 ^Bbc^	1.82 ± 0.14 ^ABbc^	1.9 ± 0.1 ^Aab^

Note: GBSs, green bamboo shoots; LBSs, lei bamboo shoots. Results followed by different capital letters in the same row indicate differences during storage. Different lowercase letters in the same column indicate differences among the samples. CK represents control samples; OA1, OA2 and OA3 represent samples treated with oxalic acid at 2.5 mmol·L^−1^, 5 mmol·L^−1^ and 7.5 mmol·L^−1^, respectively; AA1, AA2 and AA3 represent samples treated with ascorbic acid at 28.4 mmol·L^−1^, 56.8 mmol·L^−1^, and 85.2 mmol·L^−1^, respectively; CA1, CA2 and CA3 represent samples treated with critic acid at 26 mmol·L^−1^, 52 mmol·L^−1^ and 78 mmol·L^−1^, respectively; SA1, SA2 and SA3 represent samples treated with salicylic acid at 0.5 mmol·L^−1^, 1 mmol·L^−1^ and 1.5 mmol·L^−1^, respectively.

## Data Availability

The original contributions presented in this study are included in the article. Further inquiries can be directed to the corresponding author.
